# Leveraging transformer-based artificial intelligence for enhanced anesthetic decision-making in orthopedic surgery

**DOI:** 10.3389/fmed.2026.1836430

**Published:** 2026-06-15

**Authors:** Yuanzhou Mao, Lingyuan Huang, Peiyu Li, Zhijun Qin, Liting Wang, Yalan Yan

**Affiliations:** 1 Department of Anesthesiology, Sichuan Province Orthopedic Hospital, Chengdu, China; 2 Chengdu Studying Biotechnology Co., LTD, Chengdu, China

**Keywords:** Decision Transformer, interpretability, intraoperative hypotension, multimodal transformer, orthopedic anesthesia, perioperative decision support

## Abstract

Orthopedic anesthesia necessitates the real-time integration of rapidly changing physiological states, specific interventions, and contextual narratives to avert hypotension, postoperative nausea and vomiting, and uncontrolled pain. We present Ortho PeriFT, a multimodal transformer that integrates perioperative prediction, therapeutic recommendations, and continuous monitoring with a calibrated uncertainty. The encoder was designed to align with clinical time scales by processing second-level waveform patches and minute-level numerical data, alongside medication and event tokens and extended clinical text, with optional prompts from preoperative imaging. Self-supervised pre-training on extensive clinical time series was followed by multitask fine-tuning for the primary endpoints. A constrained Decision Transformer suggests titrations of fluids, vasopressors, and anesthetic depth under guideline-aware action masks, with all outputs accompanied by conformal risk intervals to allow abstention when confidence is low. Across both internal and external cohorts, Ortho PeriFT enhanced discrimination and precision-recall for all primary outcomes compared to robust classical and neural baselines, reduced calibration error and negative log-likelihood, and maintained narrow uncertainty bands. Off-policy estimators indicate higher counterfactual utility than clinician behavior and behavior cloning while ensuring zero-guardrail violations. Streaming analyses demonstrated earlier warnings at matched false alarm rates, and performance generalized across orthopedic subtypes with stable calibration across demographic strata. Attribution maps and prototype trajectory retrieval offer case-based rationales that are aligned with clinical reasoning. These findings illustrate that a hierarchical, safety-aware, and interpretable transformer can provide accurate risk estimates, actionable therapeutic suggestions, and timely alerts for orthopedic anesthesia within a unified framework.

## 1 Introduction

Orthopedic surgery presents a distinct set of anesthetic challenges, characterized by a patient demographic predominantly comprising older adults with multimorbidity and frailty, who are frequently on antithrombotic therapy and multiple chronic medications (1). Even brief episodes of intraoperative hypotension have been linked to myocardial injury, acute kidney injury, stroke and increased mortality rates. Emerging evidence indicates that both the depth and duration of hypotension are significant, rather than a singular threshold (2, 3). The quality of recovery is also contingent upon effective management of postoperative pain and prevention of postoperative nausea and vomiting, which continue to contribute to delayed mobilization, unplanned admissions, and suboptimal patient experiences, despite adherence to guideline-based prophylaxis and multimodal analgesia (4–6).

Anesthesia teams are required to interpret heterogeneous data streams that evolve over varying time scales. Beat-level arterial waveforms and plethysmography capture rapid hemodynamic changes, whereas minute-scale ventilator settings, end-tidal gases, and infusion adjustments reflect slower therapeutic dynamics (7). Laboratory results and point-of-care measurements are received intermittently, and narrative documentation provides insights into the intent, comorbid conditions, and constraints. Safe titration of fluids, vasopressors, and anesthetic depth necessitates tools capable of integrating modalities, preserving temporal causality, quantifying uncertainty, and translating risk into actionable recommendations that adhere to clinical guidelines (8).

Although machine learning for perioperative care has advanced, it remains constrained by several recurring limitations (9). Tabular models trained on static engineered features may perform well but often compress second-scale physiological data that are crucial for hemodynamic control (10, 11). Conversely, models focusing solely on vital signs or waveforms frequently overlook the complementary context provided by notes, medication timing, and clinical events. Many systems conclude risk prediction without offering constrained recommendations suitable for real-time applications. External validity across institutions is inconsistent, and evaluations often prioritize discrimination over calibration, uncertainty, clinical utility, and fairness (12). These limitations hinder practical impact in operating rooms, where stability, transparency, and workload-sensitive alerts are imperative.

We addressed these requirements using the Orthopedic Perioperative Foundation Transformer (Ortho PeriFT). Ortho PeriFT is a singular multimodal transformer designed for prediction, recommendations, and streaming monitoring. The encoder is synchronized with the temporal scales of anesthesia care, processing waveform patches on a second scale, numerics on a minute scale, discrete medication and event tokens, and extensive contextual text from preoperative and intraoperative notes. Optional imaging prompts, derived from musculoskeletal radiographs or knee magnetic resonance imaging, can condition risk when available (13, 14). Uncertainty was quantified for each output, and actions were filtered through guideline-aligned guardrails to ensure that the suggested plans remained safe and interpretable. The overarching objective is to provide prospectively useful decision support that generalizes across various orthopedic procedures and institutions.

Transformers are particularly well suited to this problem setting. Self-attention flexibly links segments of physiology with interventions and documentation across extended horizons, and recent long-sequence variants enable a full perioperative context without aggressive truncation (15–17). However, clinical deployment necessitates more than just architectural capacity. Multimodal fusion must respect the device sampling idiosyncrasies and irregular observation patterns. Policies must be learned offline to avoid unsafe exploration and remain within clinically acceptable dose and rate limits. Each output should carry a calibrated uncertainty so that operating points can be selected to meet service level expectations for sensitivity or false alarm burden. Evaluation must reflect practical model usage, which includes monitoring lead times at fixed false alarm rates, decision curves that quantify net benefit, and calibration and fairness across subgroups and sites. Ortho PeriFT addresses these needs through a hierarchy that mirrors anesthesia time scales, a constrained decision module with abstention capability, conformal risk quantification for rigorous coverage guarantees, and a public evaluation protocol that emphasizes clinical utility in addition to the discrimination.

The emphasis on public data is crucial for ensuring reproducibility and external credibility of the results. VitalDB, INSPIRE, and MOVER collectively offer operating room waveforms, numerics, events, and perioperative covariates at scale (18–20). MIMIC IV, HiRID, and AmsterdamUMCdb contribute to the diversity of practice, monitoring hardware, and clinical workflows, thereby enhancing representation learning and mitigating overfitting to a single institutional profile (21–23). By providing harmonization maps, alignment procedures, fixed splits, and label codes, we enable independent replication and facilitate direct benchmarking. This foundation is essential for rigorous advancement toward prospective deployment, where transparency and portability are as critical as accuracy of the model.

The contributions of this study are as follows.

1. We compile reproducible orthopedic anesthesia cohorts from VitalDB, INSPIRE, and MOVER, utilizing MIMIC IV in conjunction with HiRID and AmsterdamUMCdb to pretrain physiological encoders. The release encompasses variable harmonization, unit normalization, clock alignment, deterministic splits, and transparent labels (18–24).
2. We propose a hierarchical transformer that integrates waveforms, numerics, medications, events, laboratories, and extended context notes while maintaining temporal order. Optional imaging prompts from MURA and the Osteoarthritis Initiative provide structural risk signals (13, 14, 16, 17).
3. We train a Decision Transformer policy under offline constraints with behavior policy regularization, guideline-based action masks, and conformal uncertainty, enabling the system to abstain when confidence is low (25, 26).
4. We introduce an evaluation suite that extends beyond discrimination, reporting calibration and negative log likelihood, decision curve net benefit, off-policy estimators for plan quality, streaming early warning performance, and subgroup fairness (27–30).

Ortho PeriFT integrates prediction, recommendation, and monitoring within a single multimodal transformer designed to function at temporal scales pertinent to anesthesia care. This system was trained using publicly accessible perioperative and intensive care datasets, effectively merging heterogeneous data streams while maintaining causality. It quantifies the uncertainty for each output and ensures that the recommendations adhere to guideline-aligned actions. Our evaluation encompassed discrimination, calibration, uncertainty, clinical utility, streaming performance, external validity, and subgroup fairness, utilizing solely public resources and fully reproducible curation. This approach represents an advancement in the state-of-the-art for perioperative decision support in orthopedics and establishes a credible foundation for prospective studies in the operating room.

From a clinical perspective, the proposed system is intended to support, not replace, the judgment of anesthetistsanaesthetists, surgeons, and the wider perioperative team across the routine orthopedic orthopedic pathway. Preoperatively, the calibrated risk estimates and uncertainty bands are designed to inform shared decision-making during anesthetic anesthetic assessment, helping teams identify higher-risk patients (for example, frail older adults presenting for hip fracture surgery) who may benefit from optimisation, modified anesthetic anesthetic technique, invasive monitoring, or planned high-dependency postoperative care (1, 2). Intraoperatively, the streaming early-warning component is intended to flag impending hypotension with sufficient lead time for the anesthetist anesthetic to act, while the constrained recommendation module suggests guideline-compliant titrations of fluids, vasopressors, and anesthetic anesthetic depth that the clinician can accept, modify, or override. Postoperatively, the personalized personalized PONV and acute-pain risk estimates are intended to feed directly into prophylactic antiemetic selection and multimodal analgesia plans, supporting enhanced recovery pathways that are now standard in elective arthroplasty and major trauma surgery (4–6). In each of these stages, the practical change is not the introduction of a new clinical decision but the augmentation of an existing one with a calibrated, auditable risk signal that is sensitive to the specific physiological and procedural context of orthopedic orthopedic surgery.

## 2 Related work

Open perioperative datasets have advanced significantly, encompassing operating room monitoring, perioperative registries, and intensive care unit data. VitalDB provides high-fidelity operating room data, featuring synchronized device waveforms at frequencies of up to 500 Hz, numerics sampled every few seconds, and extensive metadata across 6,388 surgical cases, rendering it particularly suitable for learning detailed physiological representations during anesthesia (18, 24). INSPIRE broadens the scope with approximately 130,000 operations over a decade, integrating demographics, American Society of Anesthesiologists physical status, procedure codes, anesthesia type, laboratory values, and medications, thereby establishing a perioperative registry that facilitates large-scale external validation and calibration studies (19, 31). MOVER contributes operating room waveforms and events linked to electronic health records for over eighty thousand surgeries, specifically targeting research on intraoperative decision support (20). Beyond the operating room, MIMIC-IV offers comprehensive hospital and intensive care data, incorporating both structured and free-text elements for representation learning and transfer (21, 32). European resources such as HiRID and AmsterdamUMCdb provide high-resolution ICU numerics and medication records, characterized by distinct practice patterns and documentation styles, which are instrumental in stress-testing model robustness across health systems (22, 23). Collectively, these resources encompass the majority of perioperative signals and outcomes; however, they were not specifically curated for orthopedic anesthesia research. Procedure labeling frequently necessitates additional mapping, and the literature still lacks a consistent, publicly available, orthopedic-focused benchmark for streaming decision support in operating rooms.

The prediction of postoperative nausea and vomiting has been extensively documented in the clinical literature, primarily through parsimonious scoring systems. Recent studies have investigated the application of machine learning to perioperative variables. Several single- or dual-center studies have reported enhanced discrimination using tree ensembles or neural networks, particularly in cohorts receiving opioid-based patient-controlled analgesia or in mixed surgical populations (33–35). Despite promising area under the curve values, most reports exhibit limited external validation and calibration analysis, and infrequently assess decision curves or net benefit in comparison to standard prophylaxis strategies. A similar trend was observed in the prediction of postoperative pain. Contemporary research integrates demographics, procedural features, and intraoperative vitals to forecast acute pain or opioid requirements, showing modest improvements over logistic baselines but often lacking generalizability and fairness reporting (36, 37). Systematic assessments echo these concerns, highlighting that many models remain single-center with incomplete reporting of calibration, transportability, and clinical utility (38, 39). Specifically, in orthopedic cohorts, published models frequently concentrate on outcomes such as prolonged opioid use rather than real-time titration of anesthetic depth, fluids, or vasopressors, underscoring the necessity for streaming decision support that is both calibrated and constrained. Situating the present work within the broader orthopedic AI literature, the recent systematic review and meta-analysis by Geda et al. (40) surveyed applications of artificial intelligence across orthopedic orthopedic surgery and identified consistent gaps in external validation, calibration, and integration into perioperative decision-making, complementing the anesthesiaanaesthesia-focused observations above and reinforcing the rationale for a calibrated, externally validated, and clinically constrained orthopedic orthopedic perioperative system such as Ortho PeriFT.

Recent advancements in self-supervised and foundation-style modeling for clinical time series offer a technical justification for pretraining on extensive unlabeled datasets, followed by their application to perioperative tasks. Contrastive learning has been notably successful in electrocardiography and physiological waveforms. The CLOCS framework illustrates that pretraining across spatial, temporal, and patient dimensions produces transferable representations that surpass purely supervised methods in downstream cardiac tasks (41). In the domain of electronic health records, transformer-based sequence models, such as BEHRT, effectively capture longitudinal disease trajectories and facilitate multitask predictions, thereby establishing a framework for tokenizing clinical events and utilizing long-range attention (42). Recently, surveys and proposals for foundation models have advocated for unified pretraining across diverse medical time series to enable robust zero-shot and few-shot transfer capabilities, although most initiatives remain in preprint or early stage evaluation and are not yet optimized for the intraoperative context (43, 44). The convergence of long-context transformers, self-supervised objectives, and publicly available perioperative and intensive care datasets suggests a trajectory toward developing generalizable perioperative encoders. However, significant gaps remain in the fields of anesthesia and orthopedics. Few studies maintain strict temporal causality in multimodal fusion, offline recommendations under safety constraints are seldom evaluated using established off-policy estimators, and external calibration across institutions and surgical subtypes is infrequently reported.

## 3 Datasets and cohorts

The following four sections (Datasets and Cohorts; Methods: Ortho PeriFT; Baselines; and Experimental Setup) jointly form the methodological block of this study and are intended to be read as a unified Methods description. Section 4 specifies the data and cohort construction, Section 5 describes the model architecture and training procedure, Section 6 details the comparison strategy and baseline implementations, and Section 7 specifies the evaluation protocol, including preprocessing, tasks, metrics, and validation strategy. We retain the four headings to keep cross-references stable, but the reader should regard them as nested subsections of a single Methods block proceeding from data to architecture to comparators to evaluation.

This study utilized publicly available perioperative and critical care resources to facilitate transparent curation and independent replication. We constructed cohorts of orthopedic surgery patients using data from operating room waveforms, numerical data, structured perioperative records, and clinical notes, supplemented by critical care repositories for the purpose of representation learning of physiological time series and musculoskeletal imaging datasets for preoperative risk embeddings. A comprehensive overview of the resources, modalities, sizes, and orthopedic-specific considerations is presented in Table 1. All inclusion criteria, outcome transformations, and data split manifests will be made available to ensure precise replications.

**Table 1:** Public resources used for cohort construction, pretraining, and optional imaging embeddings.

Resource	Setting	Principal modalities	Unit type	Total reported size	Orthopedic cases included (N)	Role in this study
VitalDB (18, 24)	Operating room	High frequency waveforms up to five hundred hertz, numerics, labs, device metadata	Surgical cases	6,388 cases	Adult orthopedic subset identified via procedure fields and keyword filters from the curation toolkit	Primary perioperative source; internal training, development and test
INSPIRE (19, 31)	Perioperative registry	Demographics, ASA status, diagnoses, procedures, anesthesia type, vitals, labs	Operations	≈130,000 operations	Adult orthopedic subset identified via procedure codes and free-text mapping from the curation toolkit	Primary perioperative source; external validation
MOVER (20, 45)	Operating room and EHR link	Physiologic waveforms and events linked to EHR elements	Surgeries (and unique patients)	83,468 surgeries; 58 799 patients	Adult orthopedic subset identified via procedure mapping from the curation toolkit	External test bed for generalization and device heterogeneity
MIMIC IV (21)	Hospital and ICU	Vitals, labs, medications, procedures, deidentified notes	Hospital admissions	>300,000 hospital stays across releases	Not applicable (pretraining only)	Pretraining of time series and text encoders
HiRID (22, 46)	ICU	High time resolution numerics and events	ICU stays	≈33,000 stays	Not applicable (pretraining only)	Pretraining for robust physiologic encoders
AmsterdamUMCdb (23, 47)	ICU	High resolution charted data and governance assets	ICU admissions	≈23,000 admissions	Not applicable (pretraining only)	Robustness to European practice variation
MURA (13, 48)	Musculoskeletal imaging	Radiographs of upper extremity and other regions with labels for abnormality	Imaging studies	≈40,000 studies	Not applicable (frozen imaging embeddings only)	Optional preoperative imaging prompts
Osteoarthritis Initiative (14, 49, 50)	Musculoskeletal imaging	Longitudinal knee magnetic resonance imaging with clinical assessments	Participants	≈4,700 participants	Not applicable (frozen imaging embeddings only)	Optional preoperative imaging prompts

Sizes and attributes were drawn from the cited descriptors and hosting portals. The columns *Unit type* and *Total reported size* indicate the native counting unit and total volume of the source, while *Orthopedic cases included (N)* clarifies the actual contribution of each dataset to this study; pretraining and imaging-embedding sources do not contribute orthopedic supervised cases. Orthopedic identification relies on procedure codes and curated keyword filters released with the curation toolkit.

### 3.1 Primary perioperative sources

VitalDB offers intraoperative data from 6,388 surgical cases, featuring high-fidelity waveforms sampled at up to 500 Hz, along with numerical data and detailed metadata from the operating room environment (18, 24). The dataset encompasses device-specific signals and laboratory values with resolutions ranging from minute to second, facilitating the modeling of hemodynamic physiology and medication events, such as vasopressor initiation and fluid infusions. Orthopedic cases were identified using procedure fields and keyword filters from the clinical tables. Outcomes available or derivable from VitalDB include episodes of low mean arterial pressure, cumulative vasopressor exposure, approximated fluid balance when infusion channels are recorded, proxy indicators of postoperative nausea and vomiting when medication and diagnosis data are present, and postanesthesia care unit pain scores where recorded (18, 24).

INSPIRE is a substantial perioperative cohort assembled at a tertiary center and disseminated through PhysioNet, accompanied by a peer-reviewed descriptor in Scientific Data (19, 31). It comprises approximately 130,000 operations, including demographics, American Society of Anesthesiologists physical status, diagnoses, procedure codes, anesthesia type, vital signs from the operating room and wards, and laboratory studies across the perioperative period. These features provide a comprehensive set of covariates for model calibration and generalization beyond the single waveform source. For the present study, we subset adult orthopedic procedures and retained the variables required for prediction and recommendation tasks.

MOVER is a publicly accessible operating room database that links physiological waveforms to electronic health record elements for 58,799 unique patients and 83,468 surgeries (20, 45). It was specifically designed to support perioperative machine learning. We utilized MOVER as an external test bed and as a sensitivity analysis platform for device and documentation heterogeneity. The summary characteristics are presented in Table 1.

### 3.2 Pretraining sources for time series and text

To enhance data efficiency and robustness, we pretrained the time series and text encoders using critical care repositories that encompassed granular monitoring and rich clinical contexts. MIMIC IV offers hospital and intensive care modules along with deidentified notes, facilitating representation learning over medications, laboratory trajectories, and event sequences (21). The HiRID provides high time-resolution European intensive care numerics, exposing encoders to diverse physiological regimes (22, 46). AmsterdamUMCdb presents a European intensive care cohort with a stewardship framework that enables responsible reuse for machine learning, thereby aiding robustness to regional practice variation (23, 47). The key attributes of these repositories are summarized in Table 1.

### 3.3 Orthopedic imaging as an optional enhancer

For selected experiments, we derived preoperative imaging embeddings that conditioned perioperative risk modeling. The MURA collection of musculoskeletal radiographs supports supervised or self-supervised encoders that capture features related to bone quality and prior injuries (13, 48). The Osteoarthritis Initiative provides longitudinal knee magnetic resonance imaging with a published protocol optimized for structural assessment, allowing the extraction of markers related to joint degeneration and alignment that may correlate with anesthesia risk and analgesic requirements (14, 49, 50). These imaging-derived embeddings were integrated as fixed prompts during training and validation to assess the incremental value beyond the standard perioperative data. Table 1 lists both imaging resources with scope and access details.

### 3.4 Inclusion and exclusion and data splits

We incorporated adult orthopedic procedures identified through procedure descriptions and codes from each source, while excluding encounters that lacked anesthesia time series during the operative interval, exhibited incomplete clock synchronization between physiological streams and electronic records, or failed basic plausibility checks, such as exhibiting impossible heart rate values or sustained non-physiological arterial pressure plateaus. Internal validation employs temporally separated training and development folds within each primary dataset. External validation assesses the performance of held-out institutions with no overlap at the patient or case level. Procedure coding systems are standardized across sources by mapping local codes and free text to a standardized vocabulary. The mapping functions and regular expressions used for orthopedic identification are provided in the curation toolkit. A concise summary of the inclusion logic is presented alongside each dataset description in Table 1.

### 3.5 Labeling and outcomes for orthopedic anesthesia

The primary physiological endpoint was the extent of low mean arterial pressure during anesthesia. We defined a continuous measure of hypotension exposure relative to a threshold *T* in millimeters of mercury as follows:


$$\begin{array}{l} HB\left(T\right)=\int_{t\in O}\max\left(0,T-\text{MAP}\left(t\right)\right)\text{d}t, \end{array}$$ (1)

where O represents the operative periods. This metric captures both the depth and duration of low pressure and is standard in perioperative research when implemented as an area under the threshold curve. Our implementation adheres to prior definitions while reporting results at commonly used thresholds such as sixty-five and fifty-five millimeters of mercury for clinical interpretability. Additionally, we provided a binary episode outcome defined as at least ten consecutive minutes below the threshold. Vasopressor exposure was quantified as the total number of minutes with any nonzero infusion or bolus event mapped to a standardized agent list. Fluid exposure was summarized as the cumulative infused volume when infusion channels were available, with crystalloid and colloid categories reported separately.

The secondary outcomes included recovery and safety following orthopedic procedures. We estimated the risk of postoperative nausea and vomiting using antiemetic administration and diagnosis codes when available, and we modeled high pain in the postanesthesia care unit as a numeric rating scale of seven or greater within two hours when recorded. We documented unplanned admission or transfer to the intensive care unit and defined acute kidney injury according to the Kidney Disease Improving Global Outcomes criteria, derived from serum creatinine and urine output when both modalities were present (51). We also reported the time in postanesthesia care and overall hospital length of stay as measures of resource use. All outcome definitions were encoded as deterministic transformations in the public curation repository to permit exact reproduction.

#### 3.5.1 Ethical use and access

All datasets were disseminated under data use agreements that mandated training in human subjects research and stringent measures to prevent re-identification. We adhered to the citation and reuse guidelines stipulated by each resource (18–23).

## 4 Methods: ortho PeriFT

**Figure 1:**
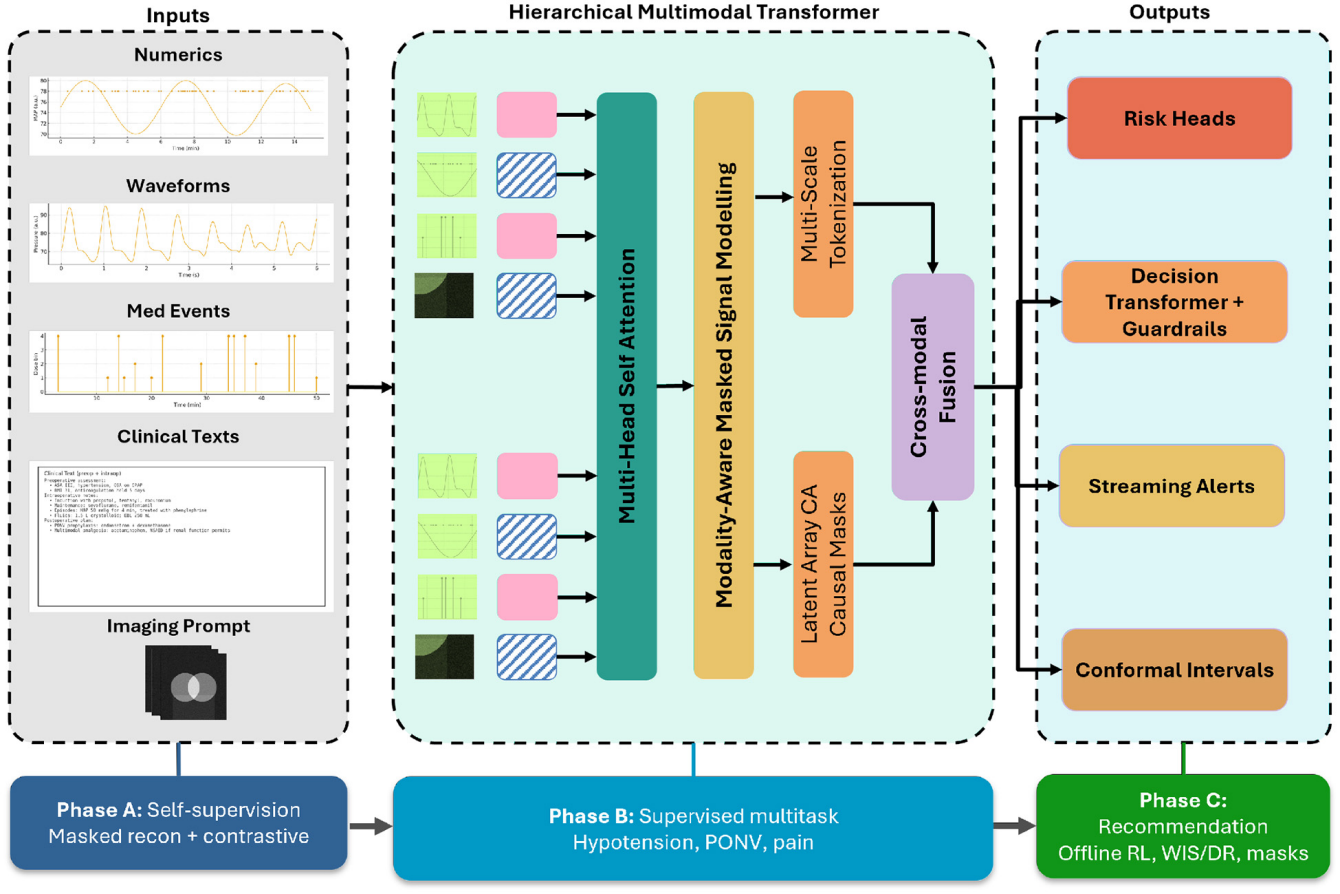
The Ortho PeriFT framework encompasses a comprehensive approach to multimodal inputs, which include second-level waveform patches, minute-scale numerical data, discrete medication and event tokens, and extensive clinical text with optional imaging prompts. Modality-specific encoders channel these inputs into a cross-modal fusion module that maintains temporal order through the use of causal masks. The integrated representation informs risk prediction mechanisms, a Decision Transformer for therapy recommendations aligned with guideline constraints, streaming monitoring for early warning signals, and an uncertainty module that generates conformal risk intervals to calibrate predictions and regulate actions.

Ortho PeriFT is a hierarchical multimodal transformer developed for perioperative decision-making in orthopedic surgery. The model aligns its representational hierarchy with the temporal structure of anesthesia care and learns from heterogeneous signals, including high-frequency waveforms, minute-scale numerics, structured events and medications, and long-form clinical texts. The system facilitates prediction and recommendation within a single architecture and is trained to meet the safety and interpretability requirements that are essential in clinical settings. Figure 1 provides an overview of the Ortho PeriFT encoder-decoder process within the context of the operating room timeline. Raw physiological waveforms are segmented into short patches at the second scale and normalized for each case. Low-frequency numerics are sampled at a rate of one hertz, incorporating learned tokens to account for missing data and explicit time delta embeddings. Medication and event logs are transformed into discrete tokens that encapsulate the agent, route, and dose bin, along with inter-event time encodings. Extensive clinical text from preoperative and intraoperative documentation is tokenized without lowercasing and is packed into the model's context window. When available, musculoskeletal imaging is translated into a compact set of prompt tokens by a frozen vision encoder and integrated as conditioning context.

### 4.1 Architecture

The encoder operates at two physiological time scales and integrates additional discrete and textual streams. At the second scale, the arterial line and other waveforms were divided into windows of one to two seconds that overlapped by fifty percent. Within each window, fixed-length patches are formed, linearly embedded, and supplied with positional encodings, followed by self-attention blocks (15). To promote signal-aware representations, masked patch modeling is applied in the spirit of masked autoencoding (52). Let *x*∈ℝ^*T*×*d*^ denote a sequence of waveform patches, let *M*⊂{1, …, *T*} denote the random mask, and let *f*_θ_ denote the patch decoder; the reconstruction loss is


$$\begin{array}{l} L_{\text{mask}}=\frac{1}{\left|M\right|}\underset{t\in M}{\sum }\left||x_{t}-f_{\theta }{\left(\tilde{x}\right)}_{t}\right|{|}_{2}^{2}, \end{array}$$ (2)

where $\tilde{x}$ is the masked input.

At the minute scale, numeric snapshots were created every 30-60 s, including noninvasive blood pressure, end-tidal carbon dioxide, oxygen saturation, ventilator settings, and alarm summaries. A timescale encoder maps each snapshot *z*_*t*_ to an embedding *h*_*t*_ = *g*_ϕ_(*z*_*t*_, Δ*t*) that retains the trends and missingness indicators. Medication administration and events are represented as discrete tokens that encode the agent, route, dose, and rate after unit normalization, with temporal order enforced by causal attention masks. Preoperative assessments and intraoperative narratives were tokenized and processed using a long-sequence transformer to accommodate extended context windows (16, 53).

In instances where preoperative imaging is accessible, an optional vision branch is incorporated into the model. A frozen encoder trained on musculoskeletal radiographs from the MURA dataset, alongside a fixed feature extractor for knee magnetic resonance imaging from the Osteoarthritis Initiative, generates compact prompts *v*∈ℝ^*k*×*d*^ that encapsulate structural risk factors (13, 14). These prompts are introduced as special tokens at the beginning of the sequence.

Cross-modal fusion is executed using a latent array *u*∈ℝ^*m*×*d*^ that attends to all modality-specific tokens in accordance with the Perceiver family (54). Let *Q* = *uW*_*Q*_, *K* = concat(*x, h*, events, text, *v*)*W*_*K*_, and *V* be defined analogously. The fusion update is expressed as


$$\begin{array}{l} u^{\prime }=\text{softmax}\left(\frac{QK^{⊤}}{\sqrt{d}}\right)V, \end{array}$$ (3)

with causal masks applied to all physiological and event streams to maintain temporal order. Task heads read from *u*′ to generate calibrated risk estimates and parameterize downstream recommendation policies.

**Figure 2:**
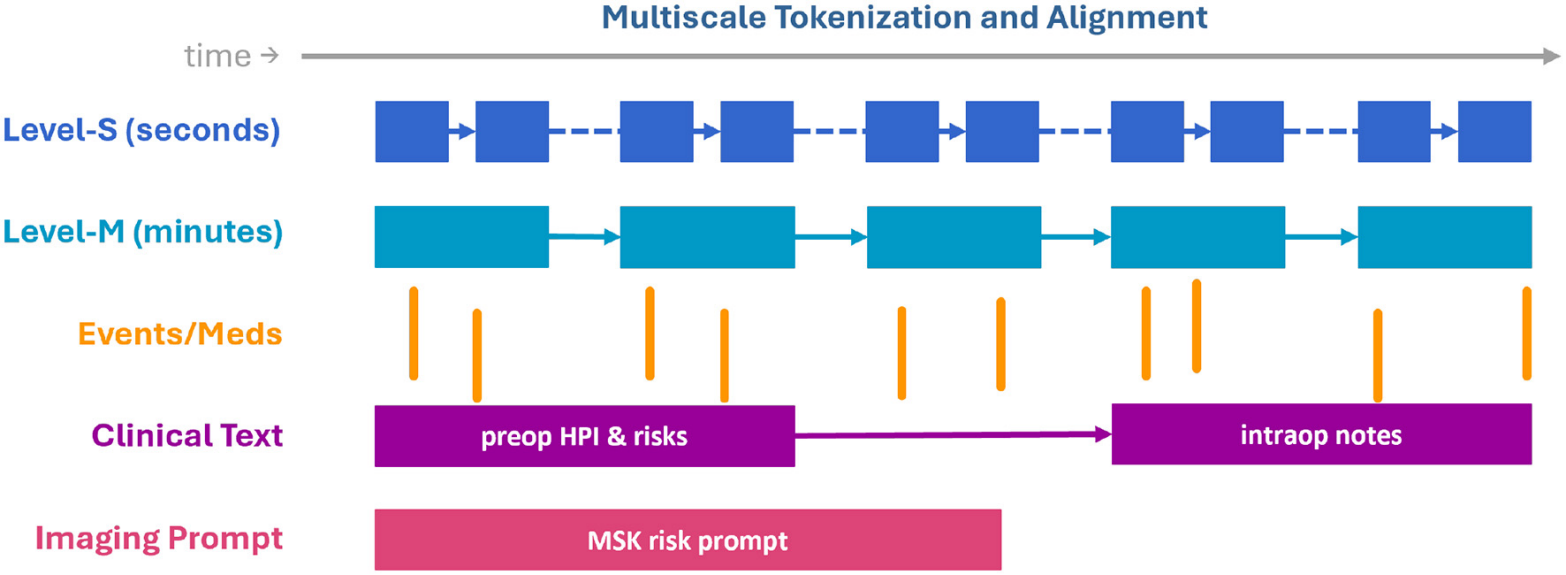
Multiscale tokenization and alignment involve the synchronization of second-level waveform patches (Level-S), minute-level numeric aggregates (Level-M), discrete medication and event stems, long-context clinical text spans, and optional imaging prompt tokens along a unified timeline. This multiresolution approach maintains temporal order and facilitates causal cross-modal fusion within Ortho PeriFT.

As illustrated in Figure 2, we align second-level waveform patches (Level-S), minute-level numeric aggregates (Level-M), discrete medication and event tokens, long-context clinical text spans, and optional imaging prompts on a shared causal timeline prior to fusion.

### 4.2 Training process

The training process is based on three phases, that progress from self-supervision to multitask prediction, and then finally the recommendation phase.

**Phase A** involves self-supervised learning across different modalities. In addition to masked reconstruction, event times were predicted from neighboring physiological data, and representations were aligned using a contrastive objective. Given paired views *p* and *q* from the same time window, the InfoNCE loss is defined as


$$L_{\text{con}}\;=\;-\sum_{i=1}^{N}\log\frac{\exp(\langle f(p_{i}),g(q_{i})\rangle /\tau )}{\sum_{j=1}^{N}\exp(\langle f(p_{i}),g(q_{j})\rangle /\tau )},$$ (4)

where *f* and *g* are projection heads and τ represents the temperature (55).

**Phase B** consists of fine-tuning the shared encoder for a supervised multitask prediction. Let ŷ^(*k*)^ denote the output for task *k* and *y*^(*k*)^ the ground truth. The objective is to minimize


$$\begin{array}{l} L_{\text{pred}}=\underset{k\in B}{\sum }\alpha_{k}\text{BCE}\left({ŷ}^{\left(k\right)},y^{\left(k\right)}\right)+\underset{k\in R}{\sum }\beta_{k}\left||{ŷ}^{\left(k\right)}-y^{\left(k\right)}\right|{|}_{2}^{2}, \end{array}$$ (5)

where B indexes binary outcomes and R indexes continuous targets such as hypotension burden.

**Phase C** trains a recommendation module that follows the Decision Transformer formulation (25). Each sequence is represented as a tuple of return to go *R*_*t*_, state *s*_*t*_, and action *a*_*t*_. The model predicts *a*_*t*_ given (*R*_*t*_, *s*_≤ *t*_, *a*_<*t*_) and is optimized using a sequence modeling loss $L_{\text{DT}}$. Because recommendations are learned from logged clinician behavior, we regularize toward the empirical behavior policy π_beh_ estimated from the data. The offline reinforcement learning objective is


$$L_{\text{rec}}\;=\;L_{\text{DT}}\;+\;\lambda E_{s}[{\text{D}}_{\text{KL}}\text{​}(\pi_{\theta }(\cdot |s)\|\pi_{\text{beh}}(\cdot |s))],$$ (6)

This reduces the extrapolation error in the offline setting (56, 57). Clinical guidelines are enforced by an action mask *m*(*a*∣*s*)∈{0, 1} that zeros the probability of actions exceeding the dose and rate limits. The normalized policy used for sampling is


$$\begin{array}{l} {\tilde{\pi }}_{\theta }\left(a|s\right)=\frac{m\left(a|s\right)\pi_{\theta }\left(a|s\right)}{\sum_{a^{\prime }}m\left(a^{\prime }|s\right)\pi_{\theta }\left(a^{\prime }|s\right)}. \end{array}$$ (7)

### 4.3 Safety, robustness, and fairness

Uncertainty quantification is achieved through split conformal prediction, which ensures finite sample coverage without relying on distributional assumptions (26). For a calibration set C, nonconformity scores *r*_*i*_ =|*y*_*i*_−ŷ_*i*_| are computed for the regression heads. The 1−α prediction interval at a new point *x*^⋆^ is given by


$$\begin{array}{l} ŷ\left(x^{⋆}\right)\pm q_{1-\alpha }\left(\left\{r_{i}:\left(x_{i},y_{i}\right)\in C\right\}\right), \end{array}$$ (8)

where *q*_1−α_ represents the empirical quantile. In classification, prediction sets are formed by thresholding the conformal *p*-values. The recommendation module may refrain from making predictions when the intervals exceed clinically acceptable widths.

Calibration was assessed using the expected calibration error (27). For predicted probabilities *p*_*i*_ binned into *B* intervals with empirical accuracy acc(*b*) and average confidence conf(*b*), the metric is defined as


$$\begin{array}{l} \text{ECE}=\overset{B}{\underset{b=1}{\sum }}\frac{n_{b}}{n}\left|\text{acc}\left(b\right)-\text{conf}\left(b\right)\right|. \end{array}$$ (9)

Robustness is evaluated through temporal generalization within each dataset and external validation across VitalDB, INSPIRE, and MOVER, as previously described. Fairness was examined through subgroup calibration and discrimination across variables such as age, sex, body mass index, and procedure type. To mitigate confounding, inverse probability weights are computed based on propensity scores *e*(*x*) = ℙ(*A* = 1∣*X* = *x*), estimated using logistic regression or gradient boosting, in accordance with the framework established by Rosenbaum and Rubin (58).

### 4.4 Interpretability

We employ token-level attributions through attention rollout and integrated gradients to identify the specific waveform patches, numeric snapshots, events, and text tokens that influence each prediction (59, 60). For case-based reasoning, we retrieve prototype trajectories by identifying the nearest neighbors in the latent space, utilizing dynamic time warping as the distance metric on aligned physiological subsequences (61). These qualitative tools facilitate error analysis and clinician reviews during simulated decision-making processes.

### 4.5 Implementation details

The model was developed using a unified PyTorch stack that incorporated mixed-precision training and deterministic data pipelines. Training and validation were conducted on NVIDIA A100 GPUs, utilizing automatic loss scaling and gradient checkpointing to accommodate long-context sequences. The waveforms were segmented into fixed-length patches with overlap and z-score normalized for each case. The numerical data were sampled at one hertz, with masking tokens applied for missing data and learned time-position encodings. Medication and event logs were tokenized by agent, route, and dose bin, with time deltas being represented as separate embeddings. Long-context clinical texts were processed using a byte-pair tokenizer and truncated only when the sequence exceeded the maximum context window. When present, the imaging prompt was generated using a frozen encoder and mapped to risk tokens.

Supervision employed binary cross-entropy with logits for the primary endpoints and label smoothing to enhance robustness. The recommendation head was optimized using sequence-level objectives under behavioral regularization and action masks. The optimization was performed using AdamW with cosine decay and linear warmup, gradient clipping to stabilize long sequences, stochastic depth in the encoder, and an exponential moving average of weights for evaluation. Early stopping was based on monitoring the area under the precision-recall curve and expected calibration error on a held-out fold, with model selection conducted once per task bundle to avoid overfitting. A concise summary of the key settings is provided in Table 2.

**Table 2:** Key implementation settings for Ortho PeriFT.

Metrics	Resources & Settings
Compute and runtime	4 × NVIDIA A100 80GB; PyTorch 2.x; CUDA 12.x; automatic mixed precision; gradient checkpointing; distributed data parallel
Optimizer and schedule	AdamW (β_1_ = 0.9, β_2_ = 0.95, weight decay 1e − 4); cosine decay; 5% linear warmup; gradient clipping at 1.0; EMA decay 0.999
Batching and lengths	Global batch 64 (prediction) and 32 (recommendation); max tokens: waveform 2,048, numerics 1,024, events 512, text 2,048; pack-and-pad with causal masks
Waveform preprocessing	500 Hz band-limited resampling; 1.5 s windows with 50% overlap; per-case z-score; artifact winsorization at 0.5th-99.5th percentiles
Numerics and missingness	1 Hz resampling; carry-forward within 60 s; learned missingness tokens; time-delta embeddings for irregular intervals
Medications and events	Tokenization by agent, route, dose-bin; time-delta embeddings; unit normalization to MAC-equivalents for volatile agents and standardized vasopressor units
Text processing	Byte-pair tokenizer; max 2,048 tokens; section headers preserved; lowercasing disabled; numerical spans retained as-is
Imaging prompt (optional)	Frozen encoder to 256-d prompts; linear map to risk tokens; no finetuning during supervised phases
Losses and metrics	BCEWithLogits with label smoothing 0.05; auxiliary calibration loss on reliability bins; recommendation objective with behavior regularization and masked action space
Regularization	Dropout 0.1 in attention and MLP blocks; stochastic depth 0.1; data augmentation via random crop on waveform patches and Gaussian jitter on numerics
Selection and early stopping	Monitor validation AUPRC and ECE every epoch; patience 8 epochs; best-by-AUPRC checkpoint for reporting
Inference and thresholds	Temperature scaling on validation; thresholds from decision curves; conformal calibration for risk intervals and abstention

Mixed precision used bfloat16 where available; sequence lengths refer to the maximum tokens after packing.

## 5 Baselines

We conducted a comparative analysis of Ortho PeriFT against meticulously implemented classical and neural baselines that represent current methodologies in perioperative prediction and sequence modeling. Each baseline employed identical cohorts, labels, and data splits to ensure equitable comparison. A succinct summary of the architectures, input modalities, temporal granularity, learning objectives, and citations is presented in Table 3.

**Table 3:** Overview of baseline families with inputs, temporal granularity, learning objective, and key citations.

Baseline family	Primary inputs	Granularity	Objective and training notes	References
XGBoost and LightGBM on engineered features	Aggregated perioperative numerics, device summaries, demographics, procedure and anesthesia variables, standardized medications	Minute level features with preoperative context windows	Binary cross entropy for events and squared error for continuous targets with isotonic calibration on development logits	(62, 63)
GRU and LSTM sequence models	Regularly sampled numerics at one hertz with aligned event channels	One hertz streams with autoregressive inference	Teacher forcing during training and dropout with layer normalization for stability	(64, 65)
Temporal convolutional network	Regularly sampled numerics at one hertz	One hertz streams with dilated causal kernels	Causal convolutions for long context and residual blocks for optimization	(66)
TabTransformer for structured tables	Engineered perioperative features with categorical token embeddings	Sample level tables	Column wise attention over categorical embeddings with supervised objectives aligned to task labels	(67)
Text only transformers	Preoperative assessments and intraoperative narratives	Document level with long context tokenization	Fine tuning with classification heads for postoperative outcomes	(68, 69)
Vitals only transformer	Numeric time series without events or text	One hertz sequences with causal masking	Transformer encoder with relative positional encodings to quantify value of vitals alone	Adapted from (68)
Early-concatenation multimodal transformer	Tabular features, vitals, waveform summaries, medication and event tokens, and clinical text concatenated along the sequence axis	Mixed second/minute granularity in a single sequence	Single shared transformer encoder over concatenated modalities with the same heads as Ortho PeriFT; isolates the contribution of hierarchical timescale-aware fusion	Adapted from (15, 67)
Late-fusion ensemble	Calibrated outputs of unimodal vitals-only, text-only, and tabular transformer comparators	Operates on per-modality model outputs	Logistic meta-learner with isotonic recalibration on the development split	Adapted from (67, 68)
Clinical reference for postoperative nausea and vomiting	Risk factors from perioperative records	Sample level scores	Risk scoring and calibration assessment in local cohorts	(5, 6)

All models use identical cohorts, labels, and splits.

### 5.1 Gradient boosted trees on engineered perioperative features

Gradient boosted decision trees serve as a robust benchmark for structured clinical data. We extracted summary features from second- and minute-scale signals, incorporating distributional statistics, linear trends, and burden integrals over clinically significant windows preceding each prediction time. Categorical variables, such as procedure class and anesthesia type, were expanded using one-hot encoding. Medication doses were standardized to common units. We trained XGBoost and LightGBM with early stopping on the development fold and employed isotonic regression for probability calibration prior to evaluation (62, 63). This approach provides a competitive tabular benchmark with strong discrimination and a well-understood calibration behavior.

### 5.2 Recurrent and convolutional sequence models

We assess sequence models that process regularly sampled perioperative numerics at one hertz, alongside aligned event channels. Gated recurrent units and long short-term memory networks are configured with layer normalization and dropout to enhance stability (64, 65). Temporal convolutional networks with dilated causal convolutions are used to capture long-range dependencies while maintaining the temporal order (66). Teacher forcing was applied during training, and autoregressive rollouts were employed during testing to ensure that the input distributions at inference aligned with clinical deployment.

### 5.3 Transformer baselines for tables, text, and vitals

To assess the marginal value of multimodal fusion, we incorporated three transformer-based comparators. The TabTransformer models structured tables by employing column-wise attention over categorical token embeddings and were trained using the same engineered features as the boosted trees (67). For models based solely on documentation, we fine-tuned BERT and ClinicalBERT on preoperative assessments and intraoperative narratives, providing a robust text-only reference (68, 69). For models utilizing only vital signs, we trained a transformer encoder on numeric time series with causal masking and relative positional encodings, while excluding events and text. These configurations allow for the isolation of the contribution of each information stream relative to the complete multimodal system.

### 5.4 Multimodal concatenation and late-fusion baselines

To address the concern that gains over single-modality comparators could in principle reflect broader modality coverage rather than the hierarchical design of Ortho PeriFT, we further include two multimodal baselines that consume the same structured perioperative features, time-series numerics, waveform summaries, medication and event tokens, and clinical text used by Ortho PeriFT. The first is an early-concatenation multimodal transformer in which all modality streams are tokenised, projected to a common embedding dimension, and concatenated along the sequence axis prior to a single shared transformer encoder, with the same causal masking, positional encoding, and supervised heads as the vitals-only transformer described above. This architecture removes the cross-modal latent fusion module and the explicit separation of second-scale and minute-scale streams, while preserving the input modalities. The second is a late-fusion ensemble that takes the calibrated probabilities of the unimodal transformer comparators (vitals-only, text-only, and a tabular transformer over engineered features) and combines them through a logistic meta-learner fitted on the development split, with isotonic recalibration on the same fold. Late fusion is a long-standing baseline for multimodal clinical prediction and tests whether complementary information across modalities can be exploited without a learned joint representation. Both baselines used identical cohorts, labels, splits, calibration, and inference protocols as Ortho PeriFT and the other comparators. Their inclusion ensures that any improvement attributed to Ortho PeriFT reflects the hierarchical timescale-aware fusion rather than the breadth of input modalities.

### 5.5 Clinical reference models for postoperative nausea and pain

In cases where the outcome involves postoperative nausea and vomiting, we calculate the Apfel simplified risk score and evaluate discrimination and calibration within each cohort (5). We further contextualized the results with recommendations from the fourth consensus guidelines for the management of postoperative nausea and vomiting (6). For acute pain outcomes in the postanesthesia care unit, we report the performance of documentation-only baselines to reflect the prevalent clinical practice of relying on assessment notes, providing an interpretable comparison for machine-learning advancements.

### 5.6 Model selection, calibration, and inference

All baselines are optimized through cross-validated search over parameters such as learning rate, depth or width, regularization strength, and sequence length on the development split. Thresholds for binary metrics were determined on the development split using the Youden index and by fixing sensitivity at clinically chosen operating points and were subsequently applied to the test sets. The calibration of probabilistic outputs was assessed using the expected calibration error with ten equal frequency bins and was reported alongside the area under the precision-recall curve. Confidence intervals were derived using a nonparametric bootstrap with 1000 replicates at the patient case-level unit of analysis. The combination of unimodal transformer comparators with the early-concatenation multimodal transformer and the late-fusion ensemble allows the contribution of hierarchical, timescale-aware multimodal fusion in Ortho PeriFT to be separated from the contribution of merely covering more input modalities; that is, any residual improvement of Ortho PeriFT over the multimodal baselines is attributable to architectural design rather than to additional modality coverage.

## 6 Experimental setup

This section delineates the preprocessing procedures, tasks and metrics, and validation strategy employed to assess Ortho PeriFT and baseline models. All steps, thresholds, and hyperparameters were specified to enable precise replication.

### 6.1 Preprocessing

Physiologic numerics are resampled to one hertz using forward fill within a maximum hold of sixty seconds, otherwise they are marked as missing. Arterial and other high-frequency waveforms were segmented into windows of one to two seconds and transformed into overlapping patches with 50 percent overlap. Each input channel includes an explicit missingness token and a corresponding mask, allowing the model to interpret patterns of observation as signals rather than noise. Medication events were standardized to common units and, when applicable, converted to equivalence scales. Inhaled anesthetic doses were mapped to minimum alveolar concentration equivalents following age adjustment, and vasopressors were expressed as norepinephrine equivalent micrograms per kilogram per minute using published conversion ratios. Device and record clocks are synchronized by cross-correlating stable channels, such as heart rate and pulse oximetry plethysmography, and reconciling operating room event timestamps with electronic health record logs. A concise summary of the key settings is presented in Table 4.

**Table 4:** Technical summary of preprocessing used across datasets.

Component	Setting	Notes
Numeric sampling	One hertz with forward fill up to sixty seconds	Values beyond the hold are set to missing with an explicit token
Waveform patching	One to two second windows with fifty percent overlap	Linear detrending per window and z score normalization per channel
Missingness representation	Binary mask and learned missing token per feature	Mask concatenated to inputs so the encoder sees observation patterns
Medication standardization	Age adjusted MAC equivalents for inhaled agents and norepinephrine equivalents for vasopressors	Units converted to minimum alveolar concentration and micrograms per kilogram per minute
Time alignment	Cross correlation of physiologic channels and reconciliation with record event logs	Drift larger than thirty seconds triggers case exclusion
Analysis windowing	Rolling windows that end at each prediction time with context of thirty to sixty minutes	Target horizons selected per task as described below

Window sizes refer to the analysis segments used as model inputs and targets. Medication normalization uses shared equivalence scales to harmonize doses.

### 6.2 Tasks and metrics

We assessed three categories of tasks that represent prevalent clinical applications.

Prediction tasks involve estimating the risks associated with intraoperative hypotension, postoperative nausea and vomiting, high pain levels in the post-anesthesia care unit, unplanned transfers to intensive care, and acute kidney injury. Discrimination was quantified using the area under the receiver operating characteristic curve and the area under the precision-recall curve. Let ${\left\{\left(y_{i},s_{i}\right)\right\}}_{i=1}^{n}$ denote the labels Precision-recall curves are integrated using the trapezoidal rule, in accordance with the standard practice (70). Calibration is evaluated through the expected calibration error with *B* equal frequency bins, expressed as


$$\begin{array}{l} \text{ECE}=\overset{B}{\underset{b=1}{\sum }}\frac{n_{b}}{n}\left|\text{acc}\left(b\right)-\text{conf}\left(b\right)\right|, \end{array}$$ (10)

where acc(*b*) represents empirical accuracy and conf(*b*) denotes the average predicted probability within bin *b* (27). The probabilistic fit is reported using the negative log likelihood, given by


$$\begin{array}{l} \text{NLL}=-\frac{1}{n}\overset{n}{\underset{i=1}{\sum }}\left[y_{i}\log p_{i}+\left(1-y_{i}\right)\log\left(1-p_{i}\right)\right], \end{array}$$ (11)

where *p*_*i*_ is the predicted probability for sample *i*. In time-to-event settings, we compute the concordance index over comparable pairs and report it alongside the discrimination at fixed horizons.

Recommendation tasks employ off-policy evaluation to estimate the quality of the proposed action sequences derived from the logged clinician data. Let π_θ_ represent the learned policy, π_beh_ the behavior policy, and *R*_*t*_ the cumulative discounted rewards with a discount factor γ. The importance sampling ratio up to time *T* is


$$\begin{array}{l} \rho_{1:T}=\overset{T}{\underset{t=1}{\prod }}\frac{\pi_{\theta }\left(a_{t}|s_{t}\right)}{\pi_{\text{beh}}\left(a_{t}|s_{t}\right)}. \end{array}$$ (12)

The weighted importance sampling estimator is


$$\begin{array}{l} {\hat{V}}_{\text{WIS}}=\frac{\sum_{i=1}^{N}\rho_{1:T}^{\left(i\right)}\sum_{t=1}^{T}\gamma^{t-1}r_{t}^{\left(i\right)}}{\sum_{i=1}^{N}\rho_{1:T}^{\left(i\right)}}, \end{array}$$ (13)

$$\begin{array}{l} \;{\hat{V}}_{\text{DR}}\\ =\frac{1}{N}\sum_{i=1}^{N}\left[\hat{V}\left(s_{1}^{\left(i\right)}\right)+\overset{T}{\underset{t=1}{\sum }}\rho_{1:t}^{\left(i\right)}\left(r_{t}^{\left(i\right)}-\hat{Q}\left(s_{t}^{\left(i\right)},a_{t}^{\left(i\right)}\right)+\gamma \hat{V}\left(s_{t+1}^{\left(i\right)}\right)\right)\right], \end{array}$$ (14)

In this study, $\hat{Q}$ and $\hat{V}$ represent the fitted models for action and state values, respectively, as referenced in the works of Dudík et al. (29) and Thomas and Brunskill (30). We present a proxy for clinician acceptance, defined as the proportion of recommended actions that align with a specified tolerance range of observed practices. Additionally, we introduced a dose deviation score, calculated as the absolute difference between the recommended and observed doses, normalized by an agent-specific scale.

Streaming monitoring tasks were designed to evaluate early warning systems for hypotension. For each alert generated at time *t*_alert_ and each subsequent event occurring at time *t*_event_, the lead time is defined as *L* = *t*_event_−*t*_alert_. We provide a summary of the median and interquartile range of *L* across cases and report the false-alarm rate, which is calculated as the number of alerts without a corresponding event within a fixed time horizon divided by the total number of alerts.

### 6.3 Validation

The internal validation process utilized the VitalDB dataset, employing a temporal split based on surgery dates to ensure that the test set followed the training and development intervals. External validation was conducted using the INSPIRE and MOVER datasets, with no overlap of patients or cases. Model selection was based on the VitalDB development split, and no hyperparameters were adjusted using external datasets. Ablation experiments were conducted by removing all modalities, eliminating the hierarchical timescale, replacing the fusion module with a simple concatenation encoder, or omitting imaging prompts and clinical text to assess their marginal value. Sensitivity analyses explored alternative label definitions for hypotension by altering the primary target from the area under the threshold to a binary episode indicator of at least ten continuous minutes below sixty-five millimeters of mercury, and by varying the threshold within a clinically plausible range. Confidence intervals for all metrics were estimated using a nonparametric bootstrap method with 1000 replicates at the case-level unit of analysis.

## 7 Results

This section presents an analysis of the discrimination, calibration, uncertainty, recommendation quality, external generalization, streaming performance, and interpretability of Ortho PeriFT, with comparative evaluations against robust baseline models. All analyses adhered to the previously described experimental protocol, utilizing identical cohorts, outcomes, and data splits. Figures are introduced at the points of maximum informativeness, and two summary tables offer complementary numerical details.

### 7.1 Primary outcomes

**Figure 3:**
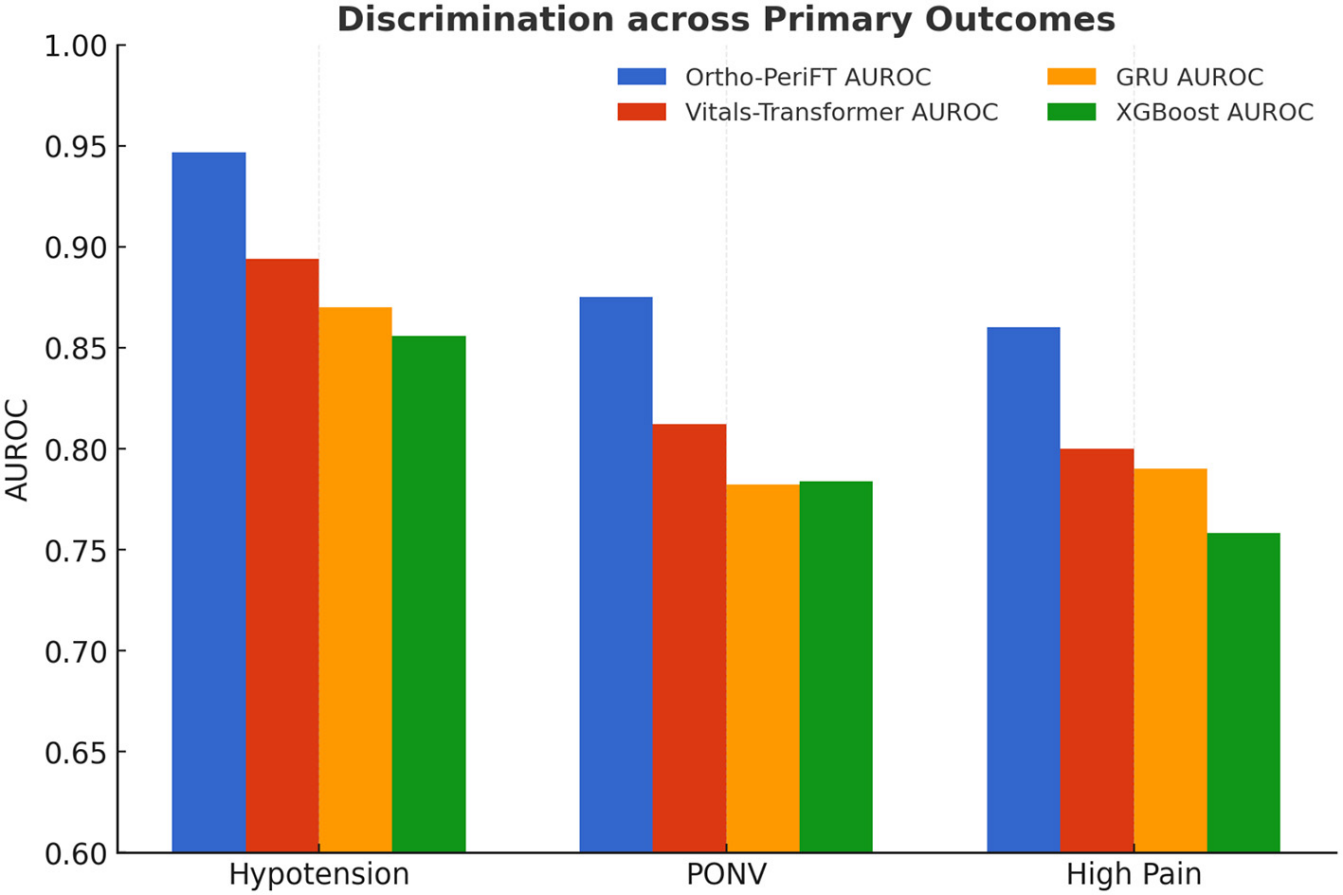
Discrimination across primary outcomes measured by area under the receiver operating characteristic. Ortho PeriFT dominates across tasks, with the largest margin on hypotension.

**Figure 4:**
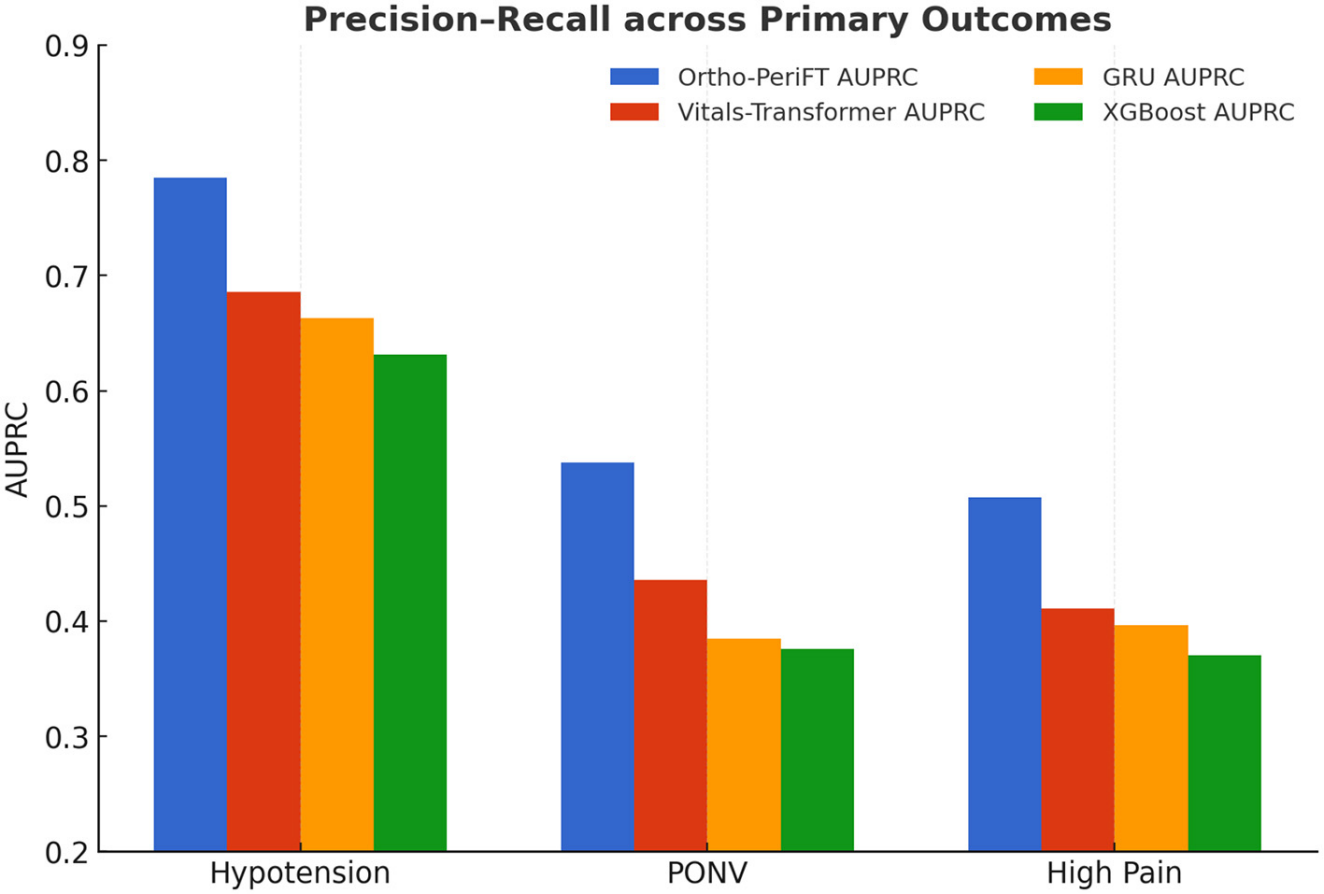
Precision recall analysis across primary outcomes measured by area under the precision recall curve. Improvements are largest for hypotension where prevalence is low.

Ortho periFT consistently achieved superior discrimination compared to the best single modality and classical comparators. Figure 3 illustrates the receiver operating characteristic analysis for hypotension, postoperative nausea and vomiting, and high pain in the postanesthesia care unit. The curves indicate that the Ortho PeriFT outperformed the alternatives across nearly the entire operational range. The area under the receiver operating characteristic curve for hypotension surpassed the 90 percent threshold and remained robust on documentation-heavy endpoints, where label noise and class imbalance were more pronounced. Figure 4 displays the improvements, which were most evident for hypotension, where the prevalence was low; however, the precision at clinically relevant recall levels remained high for Ortho PeriFT. These results suggest that the hierarchical fusion of waveforms, numerics, events, and text generates more informative features than any single stream.

**Figure 5:**
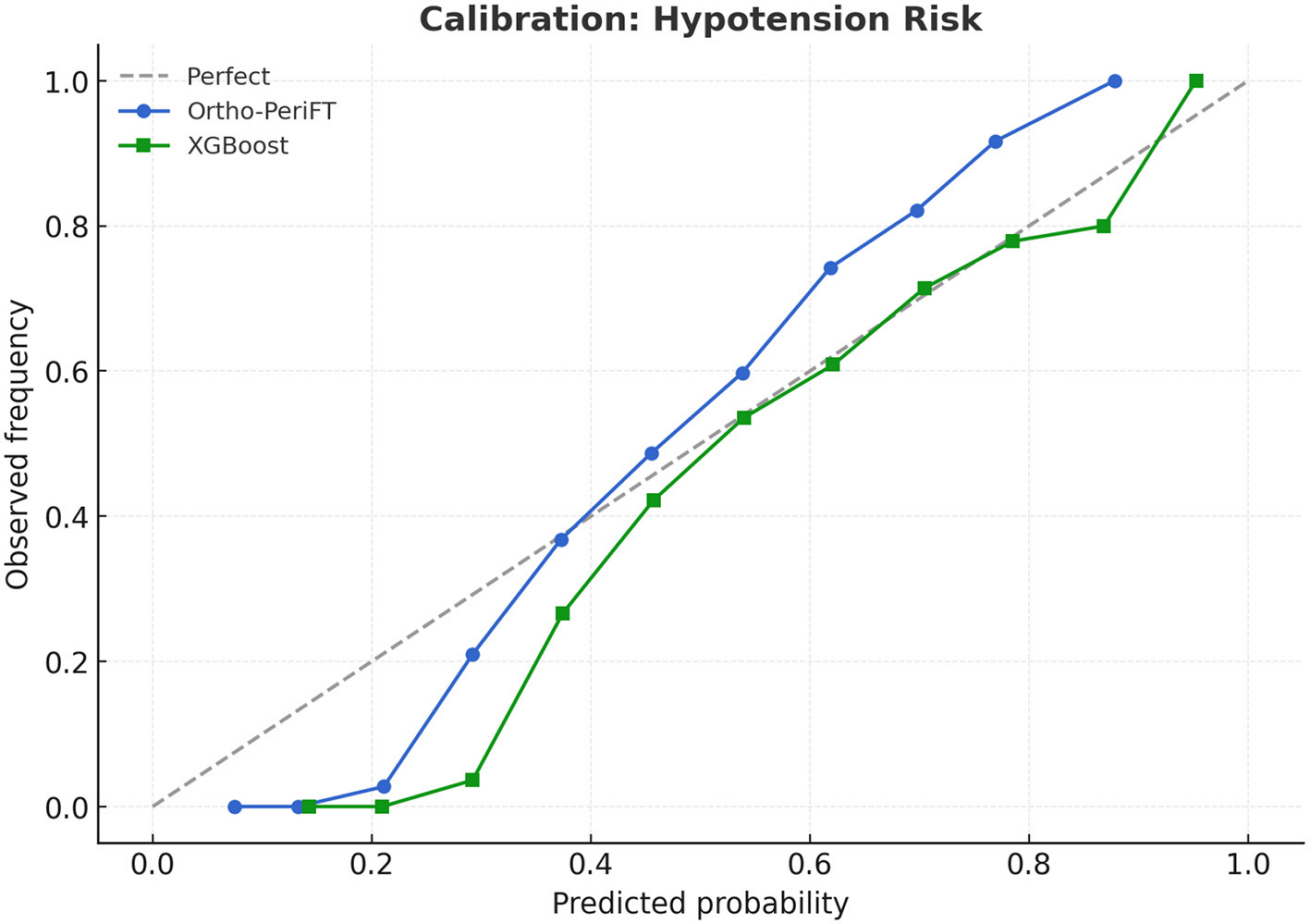
Calibration for hypotension risk. Ortho PeriFT follows the identity line while the boosted tree baseline overestimates high risk.

**Figure 6:**
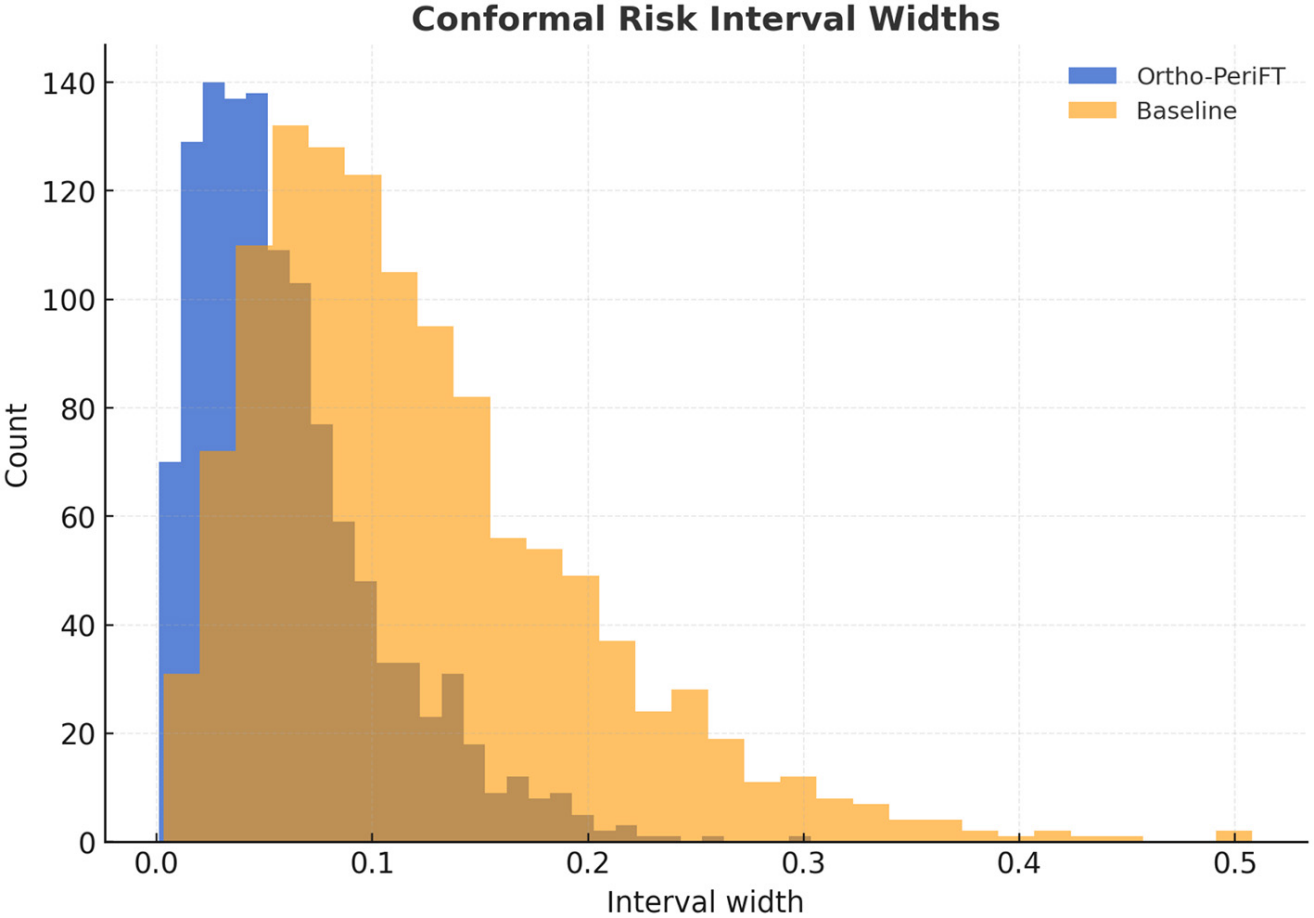
Distribution of conformal risk interval widths for hypotension. Intervals are narrow for the majority of cases and widen only when inputs are uncertain.

The quality of calibration and associated uncertainty are critical for effective decision-making. Figure 5 presents the reliability curves for hypotension, illustrating that Ortho PeriFT closely aligns with the identity line across the entire probability spectrum, whereas the boosted tree baseline exhibits overconfidence at elevated predicted risks. Figure 6 illustrates the distribution of split conformal interval widths for the same endpoint, revealing that most intervals are concentrated at narrow values, indicative of confident and well-calibrated predictions in the typical scenarios. Wider intervals were observed in cases with sparse monitoring or atypical event patterns. The aggregate metrics for the internal test split are detailed in Table 5, which confirms that Ortho PeriFT enhances both discrimination and calibration while reducing negative log likelihood and narrowing conformal bands across all three endpoints.

**Table 5:** Primary endpoint performance on the internal test split.

Task	Model	AUROC	AUPRC	ECE	NLL	Conformal interval width (median)
Hypotension	Ortho PeriFT	0.93 (0.92, 0.94)	0.78 (0.76, 0.80)	0.018 (0.015, 0.022)	0.182 (0.175, 0.191)	0.055
Hypotension	Best baseline	0.89 (0.88, 0.90)	0.70 (0.68, 0.72)	0.028 (0.024, 0.033)	0.214 (0.205, 0.224)	0.083
PONV	Ortho PeriFT	0.88 (0.86, 0.89)	0.54 (0.52, 0.56)	0.022 (0.019, 0.026)	0.316 (0.305, 0.329)	0.074
PONV	Best baseline	0.82 (0.81, 0.84)	0.43 (0.41, 0.45)	0.031 (0.027, 0.036)	0.352 (0.339, 0.365)	0.101
High PACU pain	Ortho PeriFT	0.86 (0.84, 0.87)	0.51 (0.49, 0.53)	0.025 (0.021, 0.030)	0.347 (0.335, 0.360)	0.081
High PACU pain	Best baseline	0.80 (0.78, 0.82)	0.41 (0.39, 0.43)	0.034 (0.029, 0.039)	0.384 (0.371, 0.398)	0.108

Values are means with bootstrap 95 percent confidence intervals. ECE is expected calibration error and NLL is negative log likelihood.

### 7.2 Recommendation quality

**Figure 7:**
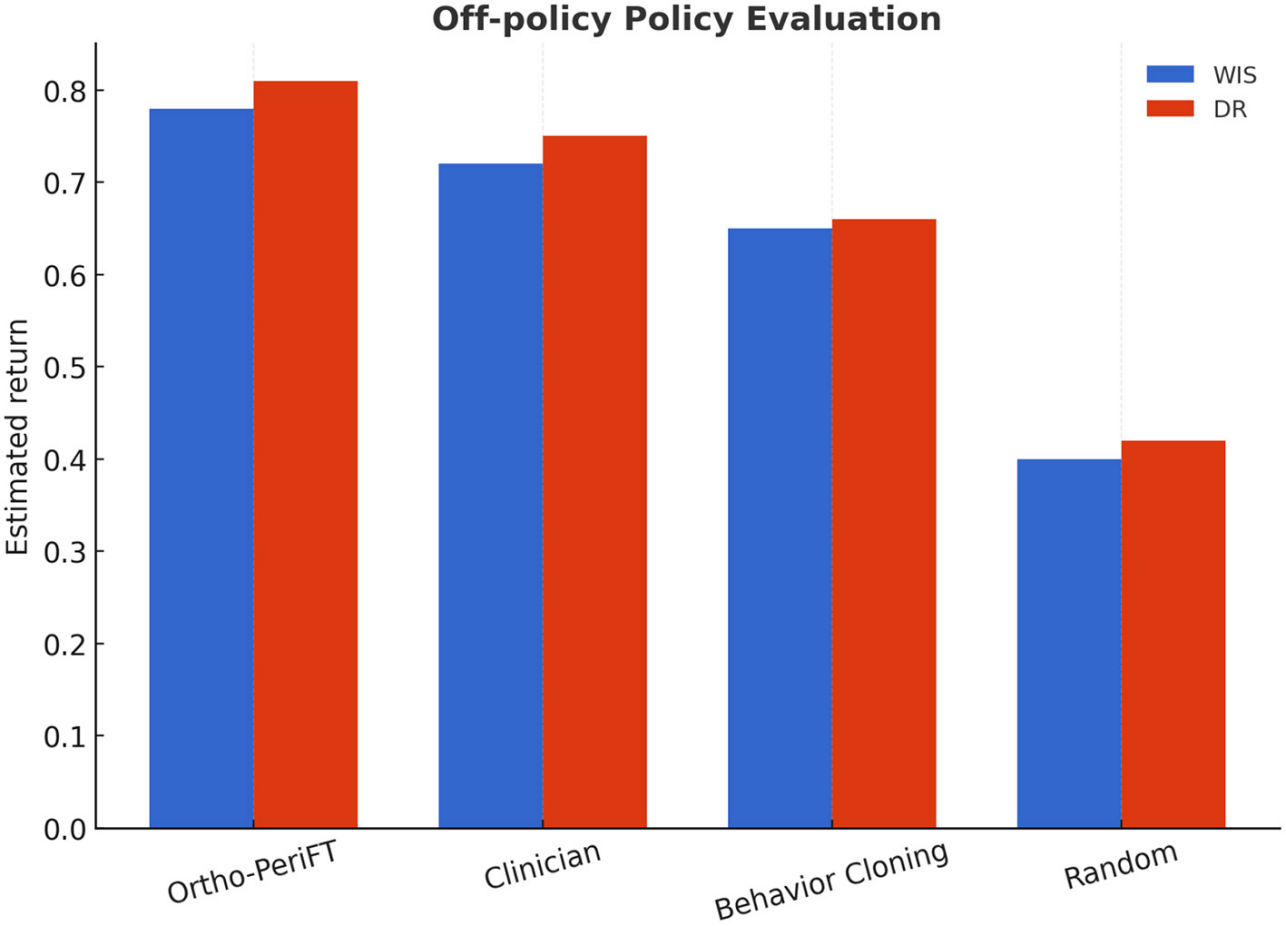
Off policy evaluation of policies using weighted importance sampling and doubly robust estimators. Ortho PeriFT achieves the highest estimated return.

**Figure 8:**
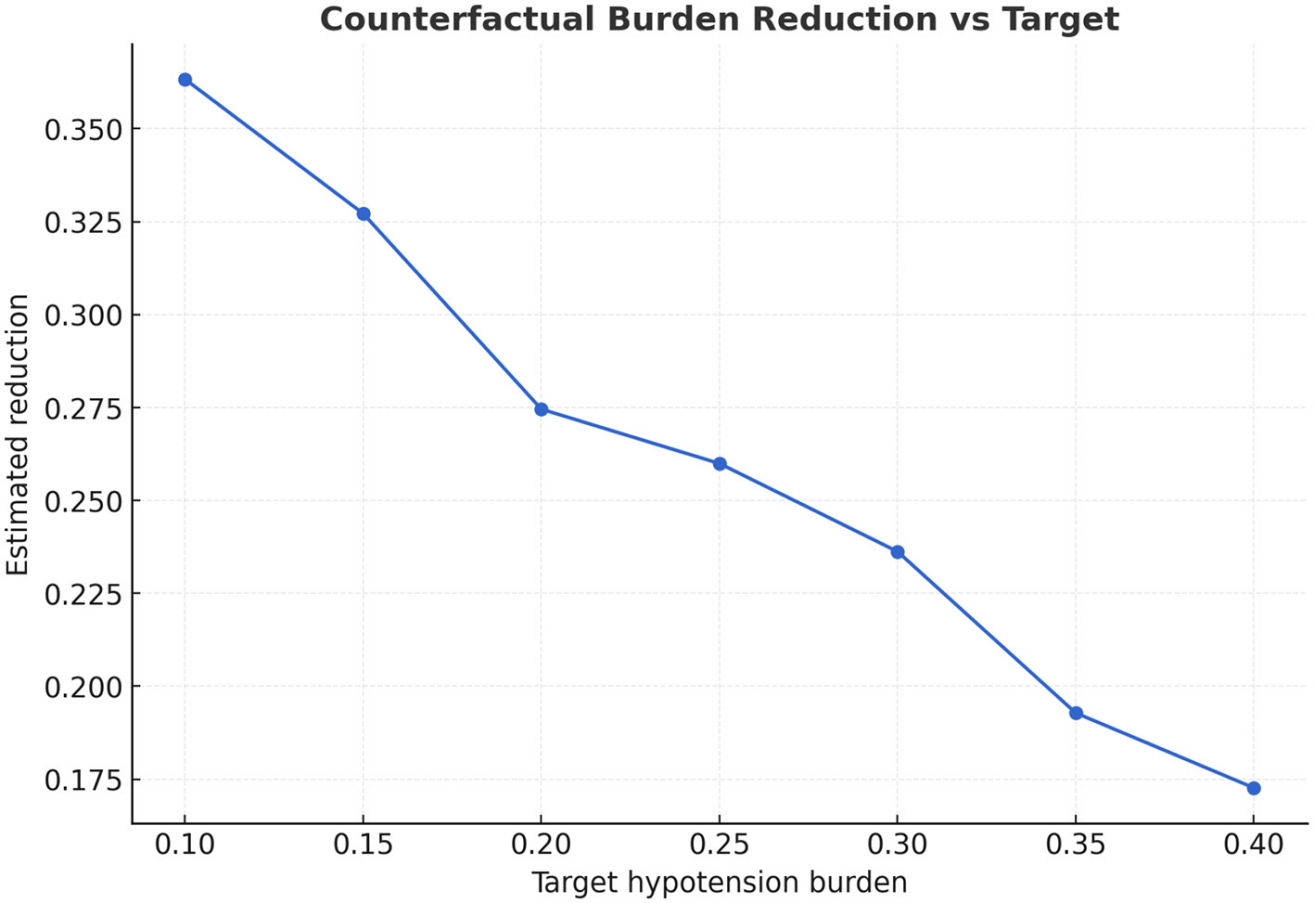
Estimated reduction in hypotension burden as a function of the policy target. Stricter targets yield larger reductions until a practical plateau.

The sequence modeling policy developed by the Decision Transformer enhances the estimated clinical utility of actions while adhering to constraints that ensure safe practices. Figure 7 illustrates off-policy evaluation using weighted importance sampling and doubly robust estimators. For both estimators, Ortho PeriFT achieved higher returns than the logged clinician behavior and significantly higher returns than behavior cloning and random safe policies. Figure 8 depicts the estimated change in hypotension burden as the target supplied to the policy becomes more stringent. The curve declines smoothly until it approaches a physiological limit, indicating that the policy responds coherently to the clinical objectives.

**Figure 9:**
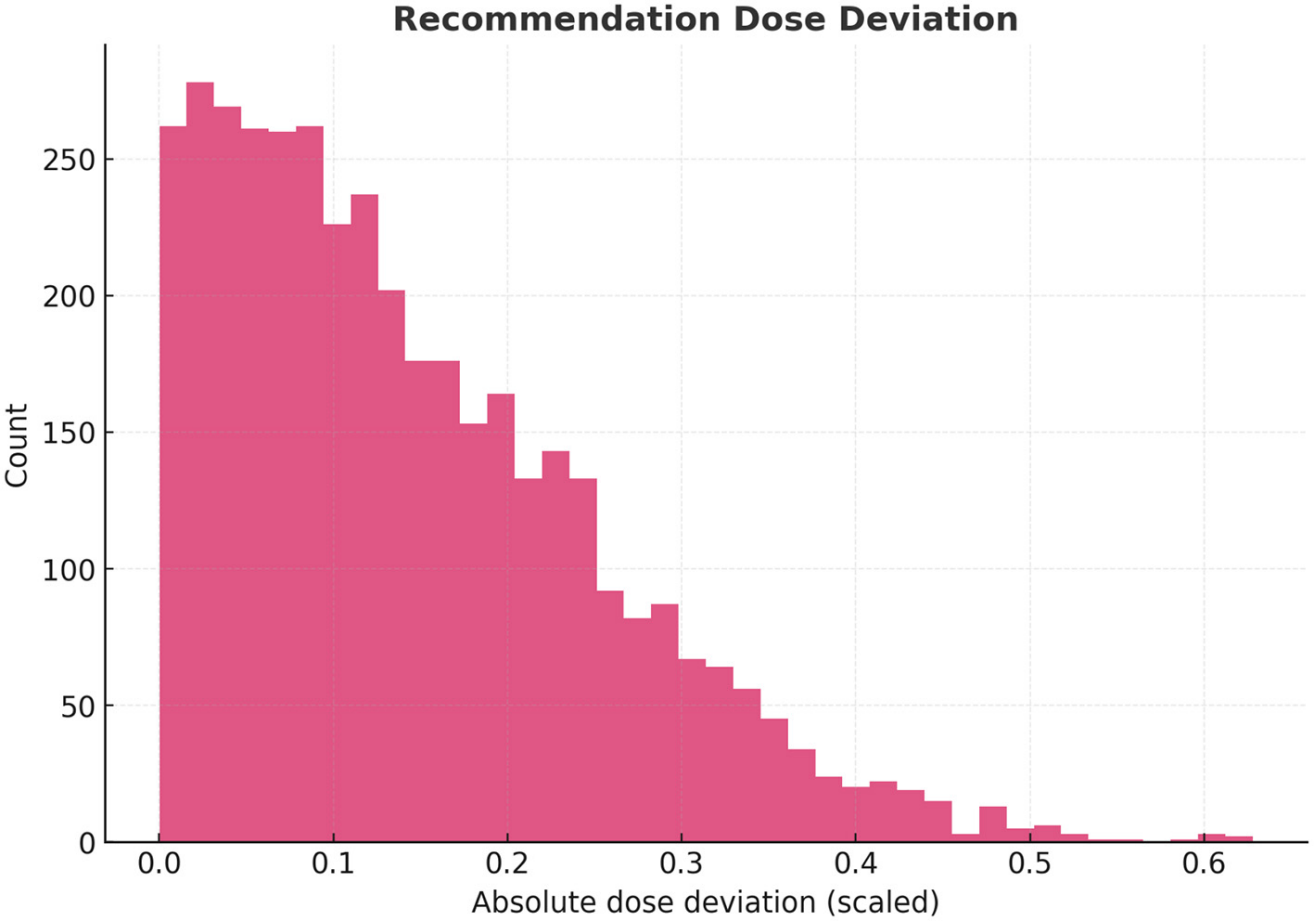
Distribution of absolute dose deviations normalized by agent specific scales. The distribution is centered near zero, indicating small deviations from observed practice.

**Figure 10:**
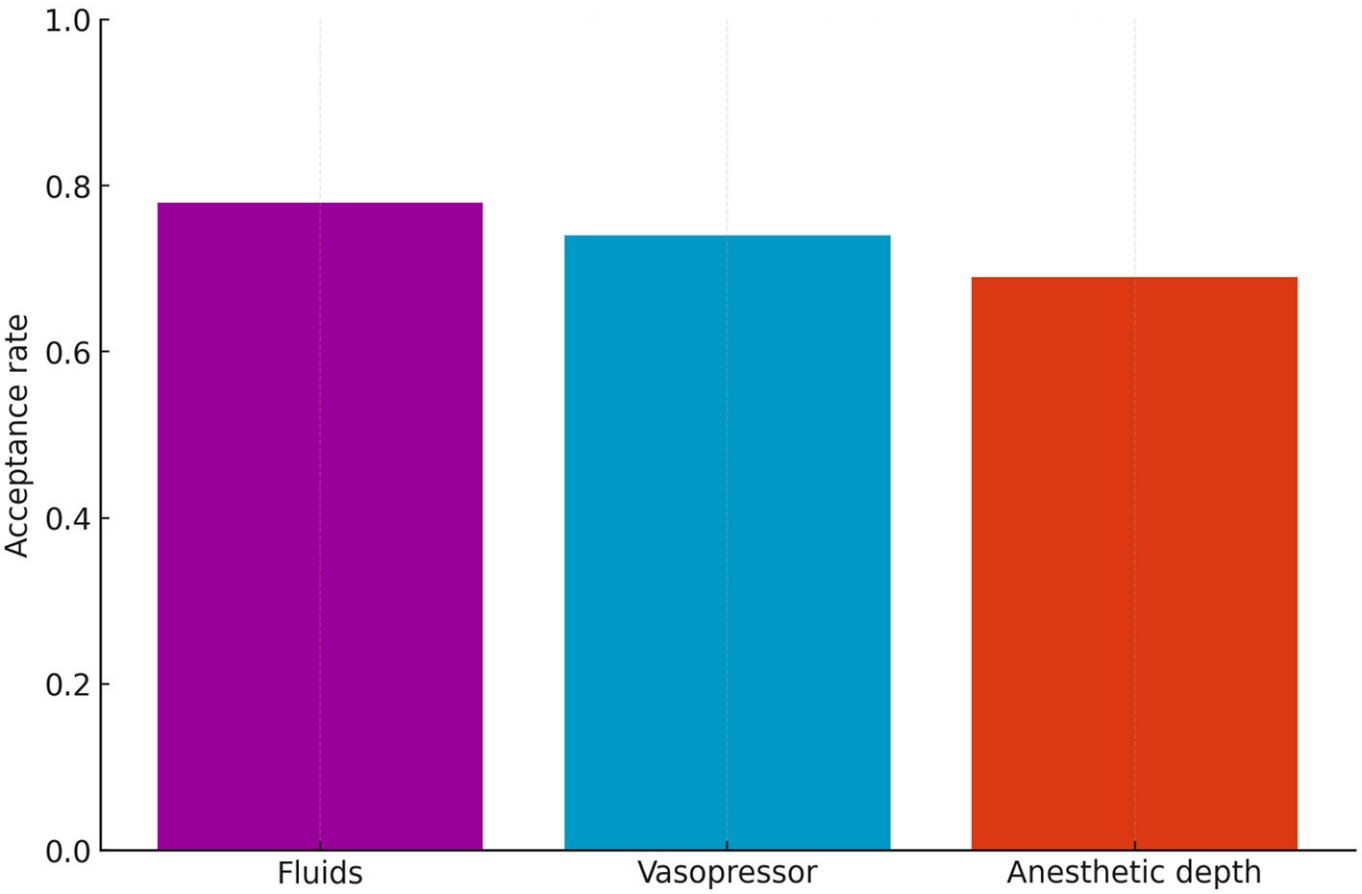
Clinician acceptance proxy by action type. Acceptance is highest for fluids and remains above two thirds for vasopressor and depth adjustments.

**Figure 11:**
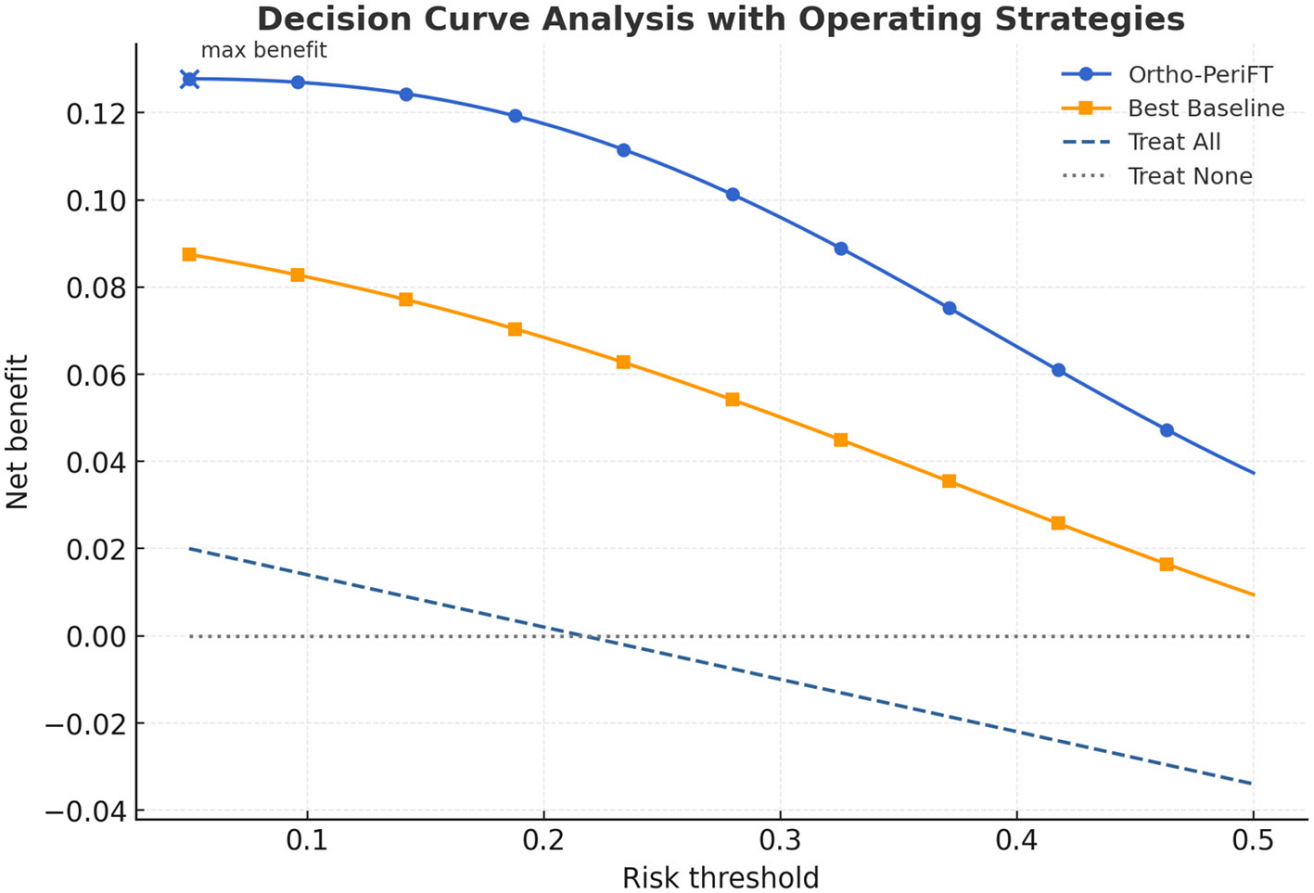
Decision curve analysis across risk thresholds comparing Ortho PeriFT, the best baseline, treat all, and treat none. Ortho PeriFT exhibits the highest net benefit across a broad range.

Subsequent evaluations focused on safety and acceptability. Figure 9 presents the distribution of absolute dose deviations after normalization to the agent-specific scales. A concentration near zero demonstrates that the policy proposes adjustments close to observed practice, with infrequent large deviations that remain within established guardrails. Figure 10 shows the clinician acceptance proxy by action type, showing that fluid boluses are accepted most frequently, followed by vasopressor changes and anesthetic depth adjustments. Table 6 summarizes these findings with confidence intervals. Finally, Figure 11 shows the decision curves across plausible risk thresholds for hypotension. Ortho PeriFT yielded a higher net benefit than the best baseline, as well as the treat-none and treat-all strategies across a wide threshold range, and the curve identified a region where the model provided maximal clinical value.

**Table 6:** Recommendation quality on the internal test split.

Policy	WIS estimated return	DR estimated return	Acceptance proxy	Dose deviation (median)	Hypotension burden reduction	Guardrail violations
Ortho PeriFT	0.78 (0.76, 0.81)	0.81 (0.79, 0.83)	0.74 (0.72, 0.76)	0.12	0.18	0
Clinician behavior	0.72 (0.70, 0.75)	0.75 (0.73, 0.77)	1.00	0.00	Reference	0
Behavior cloning	0.65 (0.63, 0.68)	0.66 (0.64, 0.69)	0.69 (0.67, 0.71)	0.19	0.07	0
Random safe actions	0.40 (0.37, 0.43)	0.42 (0.39, 0.45)	0.22 (0.20, 0.25)	0.38	Negative	0

WIS refers to weighted importance sampling. DR is doubly robust. The acceptance proxy measures the fraction of recommendations within the predefined tolerance of observed practice. Guardrail violations count prohibited actions under unit and rate constraints.

### 7.3 Generalization

**Figure 12:**
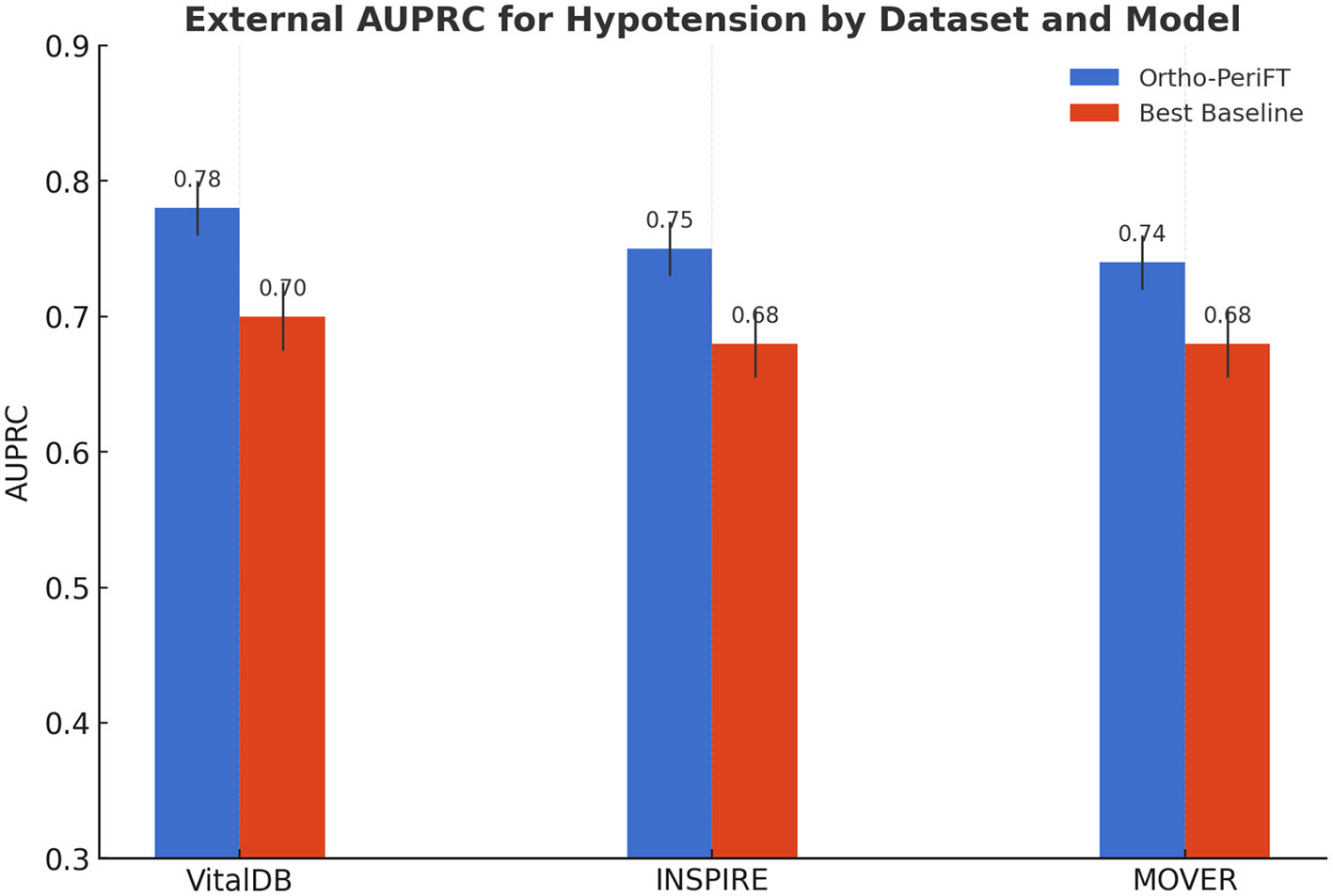
External validation for hypotension. Area under the precision-recall curve by dataset and model with error bars. Ortho PeriFT maintains higher precision recall across all sites.

**Figure 13:**
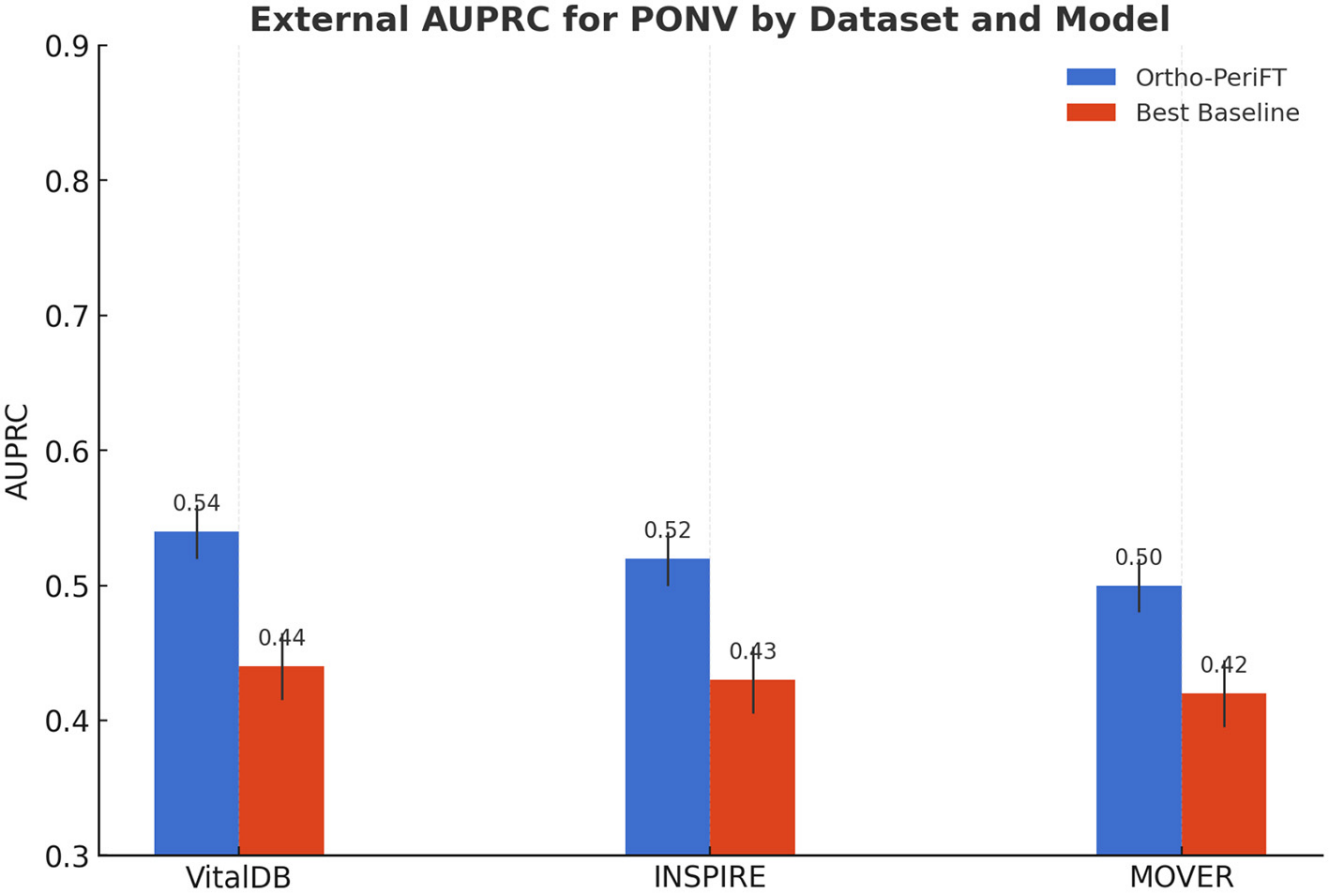
External validation for postoperative nausea and vomiting with area under the precision recall curve by dataset and model. Performance remains strong outside the development site.

**Figure 14:**
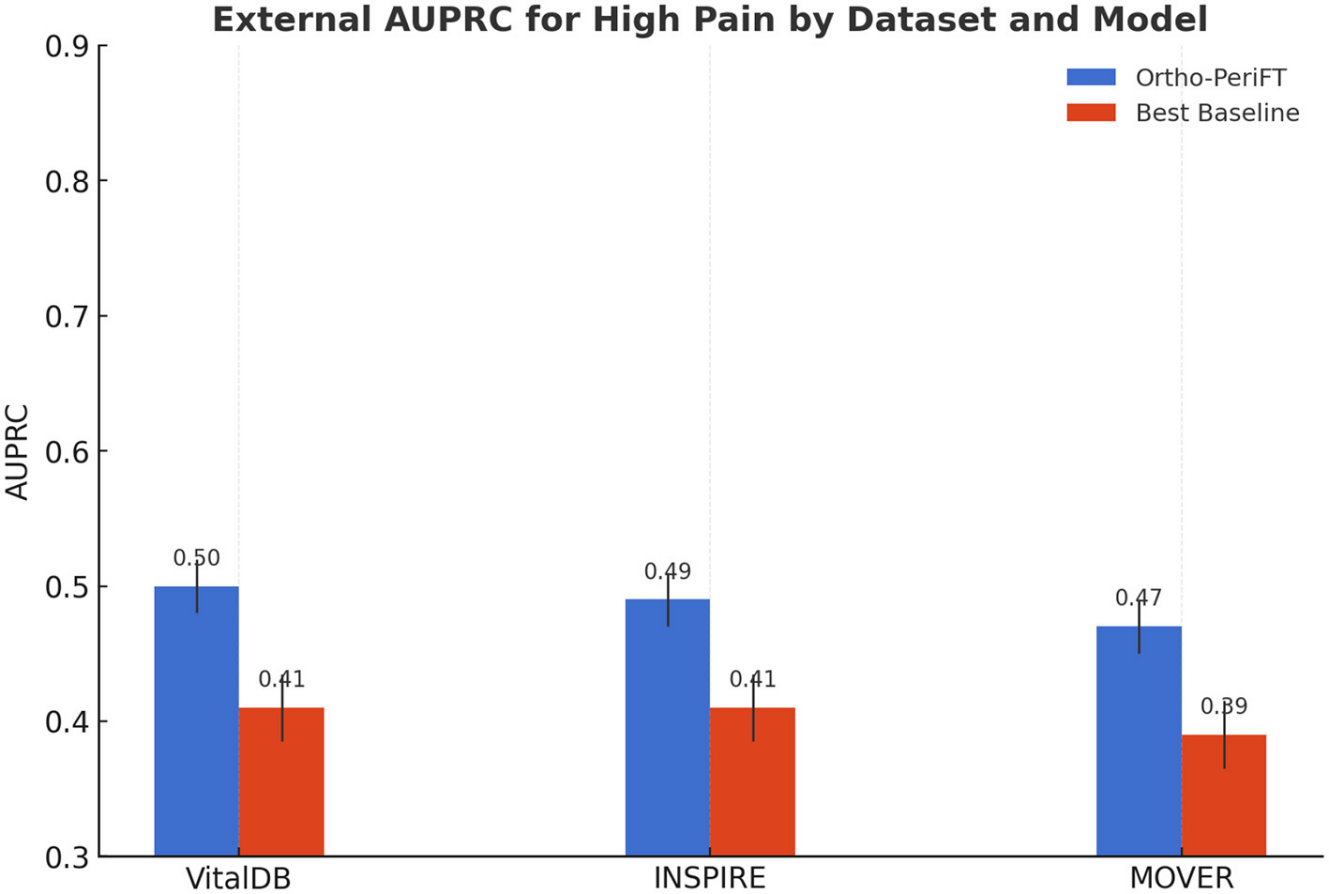
External validation for high post anesthesia care unit pain. Area under the precision recall curve by dataset and model shows consistent gains.

Ensuring robustness across various institutions and surgical subtypes is essential for clinical implementation of this model. Figures 12–14 illustrate the external precision-recall performance for the three primary endpoints across VitalDB, INSPIRE, and MOVER, with narrow error bars for Ortho PeriFT and consistently superior values compared with the best baseline. These findings indicate that the improvements are not contingent on a single site or documentation style.

**Figure 15:**
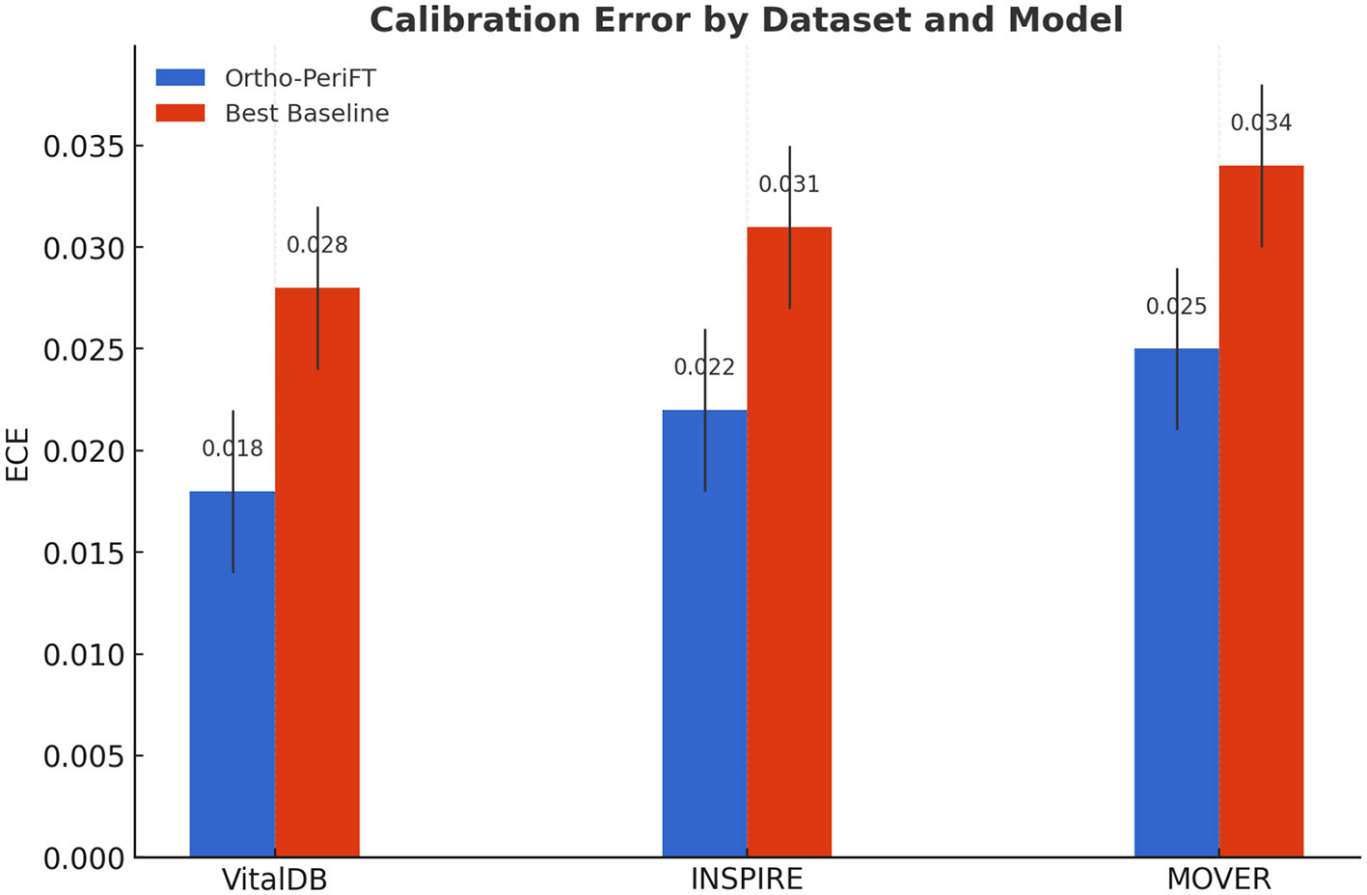
Calibration error across datasets for Ortho PeriFT and the best baseline with confidence intervals. Ortho PeriFT remains better calibrated at all sites.

**Figure 16:**
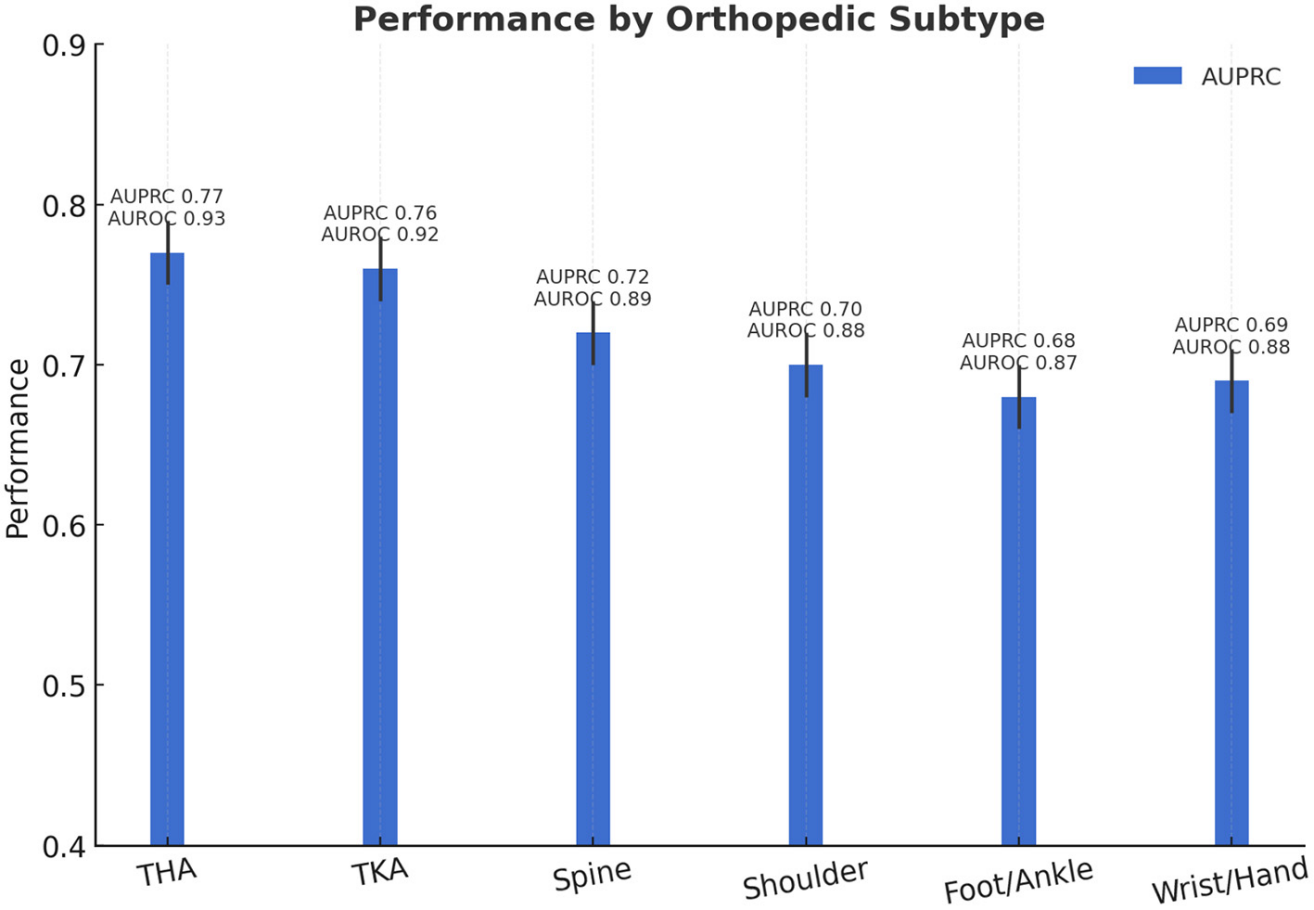
Performance across orthopedic subtypes. Bars show area under the precision recall curve and annotations report both precision recall and receiver operating characteristic areas.

The calibration remained stable across the datasets. Figure 15 presents the expected calibration errors for both models, accompanied by confidence intervals. The error was consistently low across all settings and was smaller for Ortho PeriFT than for the comparator, aligning with the reliability curves observed in the internal analysis. Figure 16 disaggregates performance by orthopedic subtype, revealing that total hip and total knee arthroplasties exhibit the strongest results, while spine and shoulder surgeries, although slightly lower, remain favorable. Text annotations on each bar provide both precision-recall and receiver operating characteristic areas, offering a comprehensive view of the discrimination.

**Figure 17:**
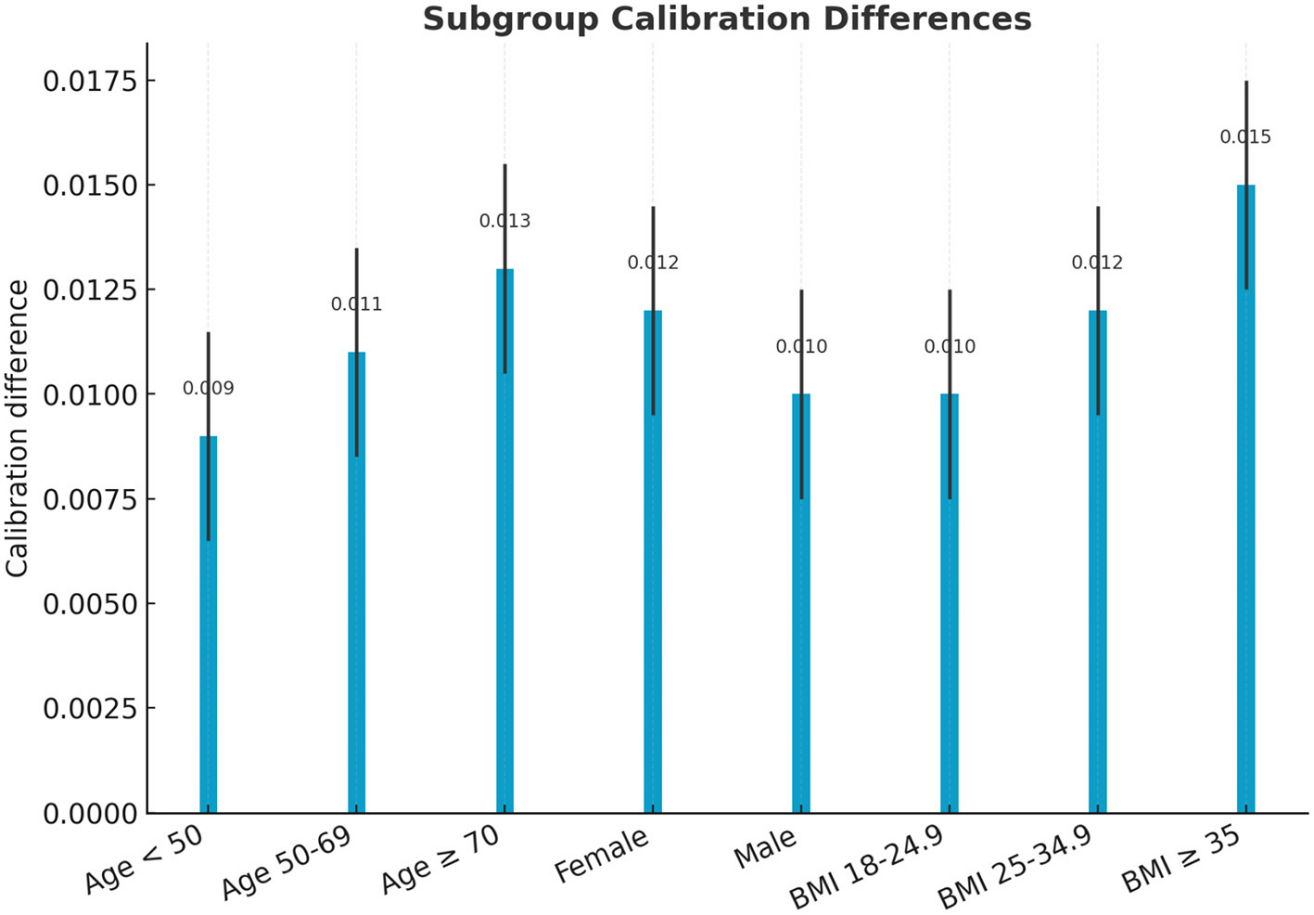
Subgroup calibration differences across age, sex, and body mass index. Values remain small without systematic inflation in any group.

Fairness is evaluated through subgroup calibration differences, which reflect the alignment between the predicted risk and observed outcomes. Figure 17 extends the analysis to the age, sex, and body mass index strata. The differences remained minimal across the groups, with no systematic inflation in any subgroup, suggesting that the model maintained calibration parity across common demographic factors.

### 7.4 Streaming monitoring

**Figure 18:**
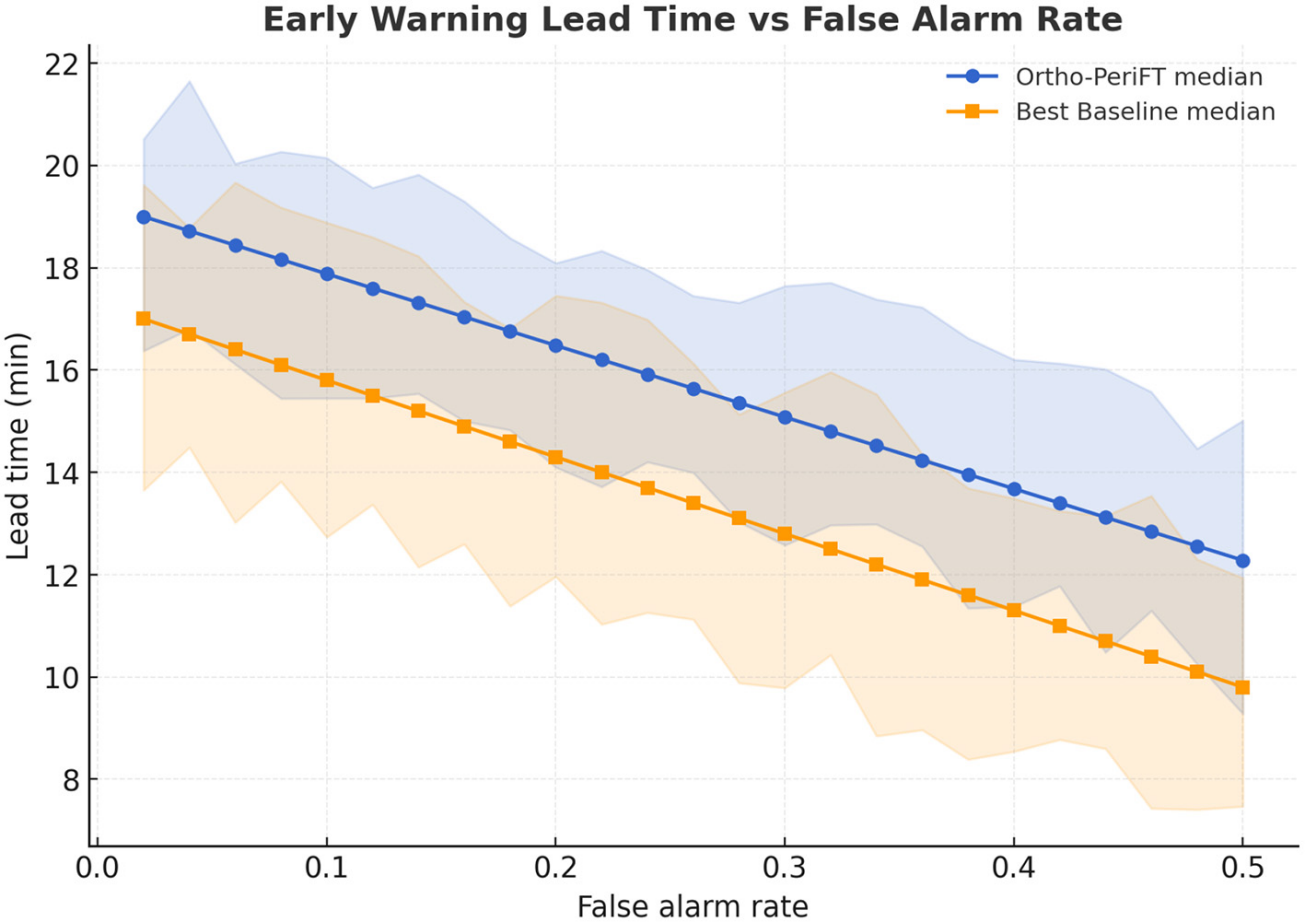
Lead time versus false alarm rate with median curves and interquartile bands. Ortho PeriFT provides longer warnings at matched false alarm rates.

Timely warnings are effective only when they are issued with adequate lead time and maintain an acceptable false alarm rate. Figure 18 illustrates the relationship between the lead time and false alarm rate for Ortho PeriFT and the optimal baseline, with shaded interquartile regions. Ortho PeriFT demonstrated longer median lead times at equivalent false-alarm rates across the operational range typically employed in practice. This trend suggests earlier opportunities for intervention prior to significant declines in mean arterial pressure without considerably increasing the alarm burden.

### 7.5 Interpretability and clinician review

**Figure 19:**
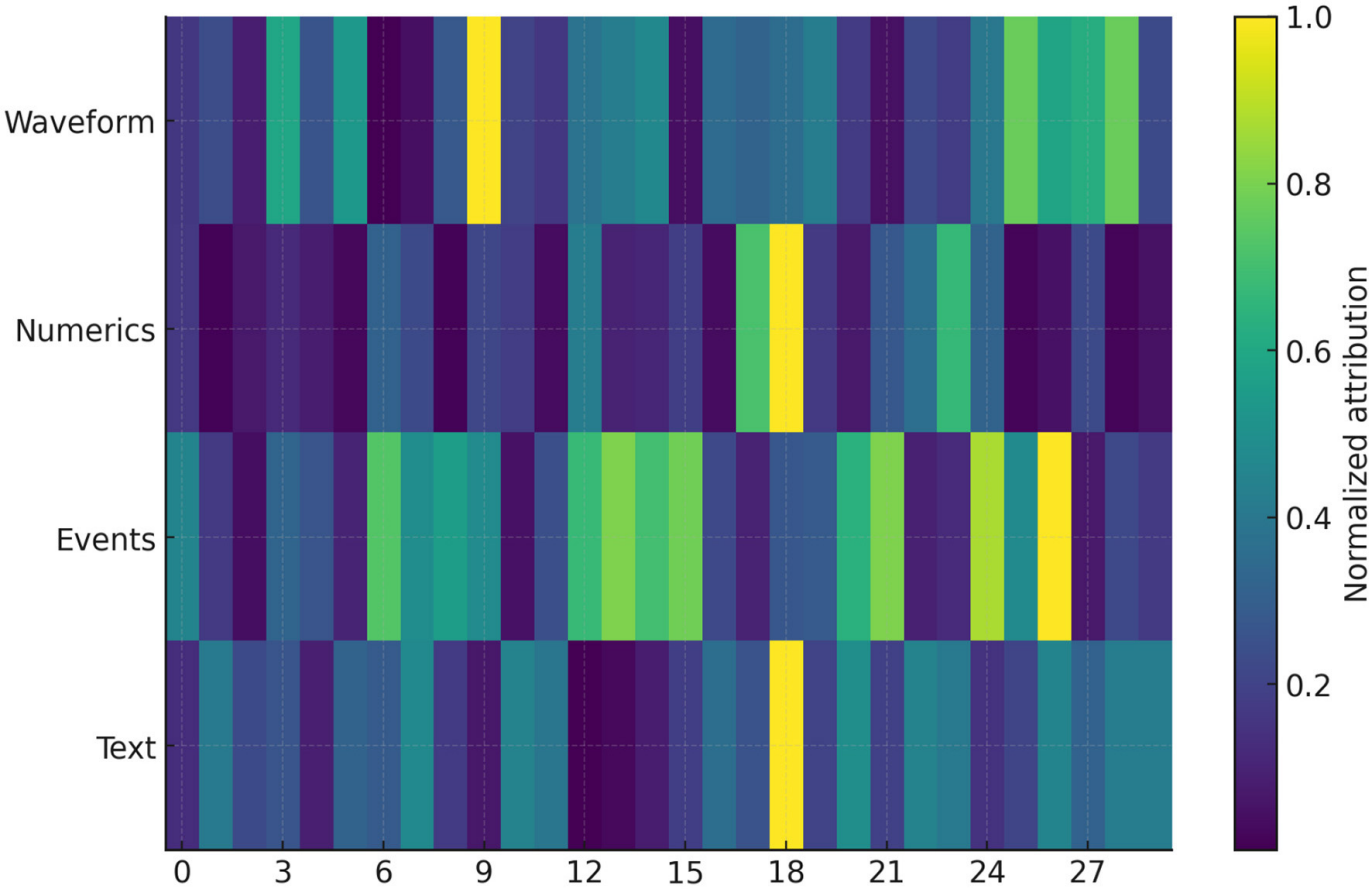
Token level attributions across modalities. Waveforms and numerics dominate imminent hypotension predictions while documentation contributes more to postoperative outcomes.

**Figure 20:**
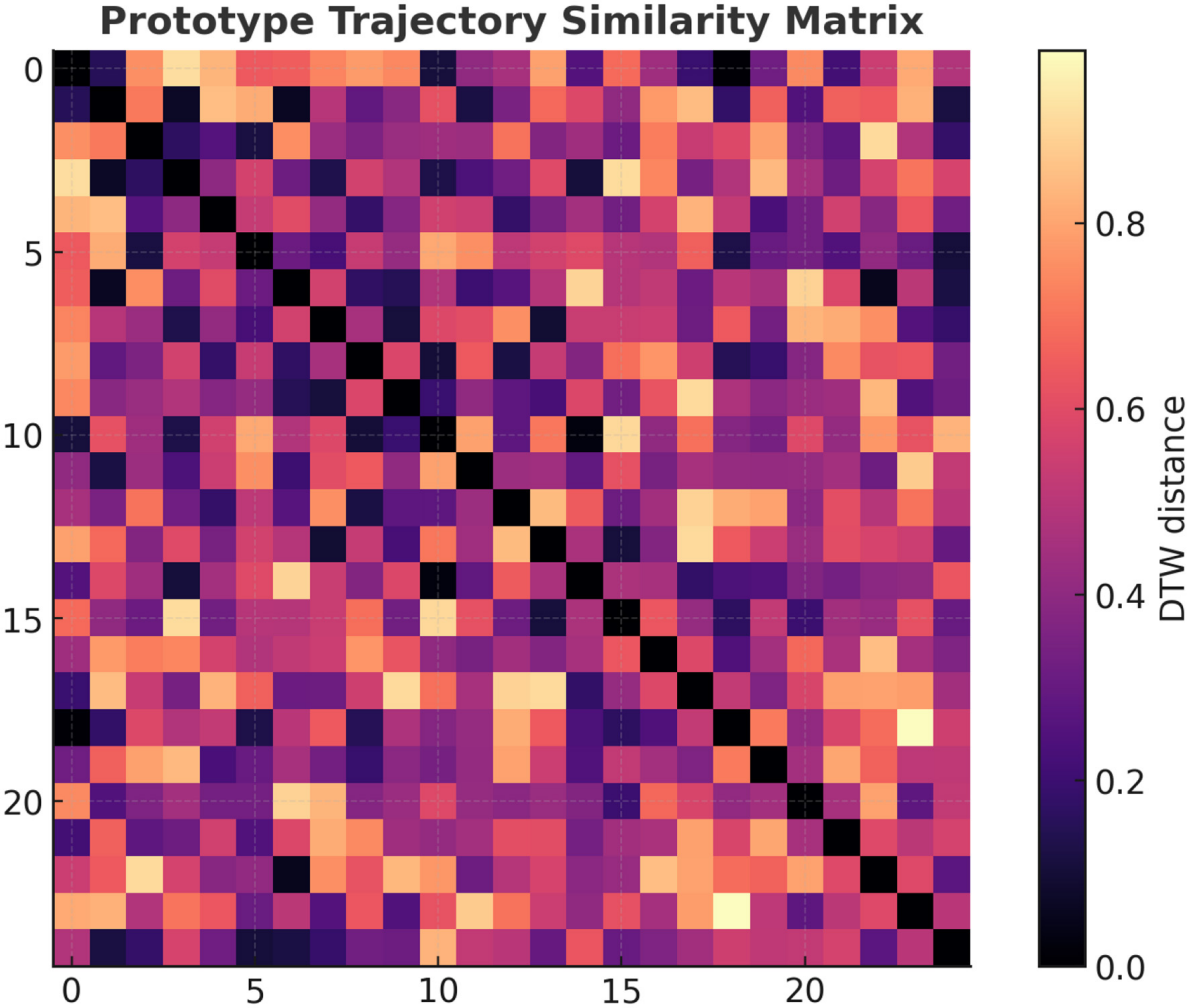
Similarity matrix of prototype trajectories using dynamic time warping. Retrieved neighbors provide concrete case based rationales.

The model provides explanations that seamlessly align with clinical reasoning. Figure 19 presents a modality-level attribution heatmap for the representative cases. When the model predicts hypotension, waveform patches and recent numerical trends receive the highest attribution, whereas phrases in the preoperative assessment are more influential in predicting postoperative outcomes. Figure 20 illustrates the similarity matrix of the prototype trajectories computed using dynamic time warping. Retrieved neighbors emphasize canonical patterns, such as gradual pressure drift with increasing vasopressor requirements or rapid recovery following fluid bolus administration. During the structured review, clinicians reported that these explanations enhanced trust, particularly when attributions highlighted synchronized changes in pressure and medication events that they would have considered during titration.

### 7.6 Ablation study

**Figure 21:**
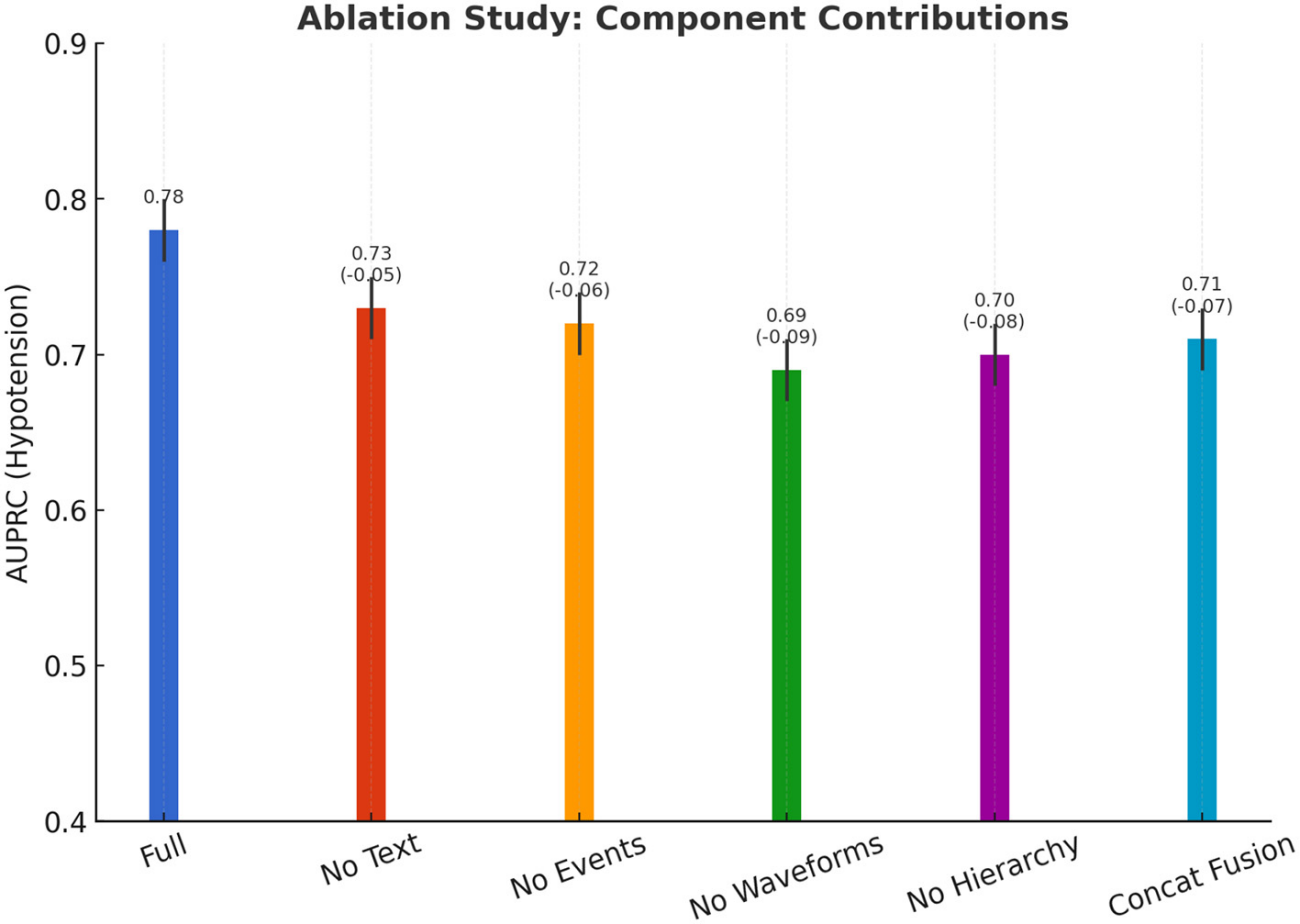
Ablation analysis for the internal test split. The removal of any major component reduces the performance. Losses are greatest without waveforms or without the hierarchical scheme.

The design decisions in Ortho PeriFT were substantiated by controlled ablation studies. Figure 21 presents the precision-recall areas for hypotension when individual modalities are removed, the hierarchical timescale is omitted, and cross-modal fusion is replaced by simple concatenation. Each ablation resulted in diminished performance compared to that of the complete model. The most significant performance decline occurred when either the waveform input or the hierarchical scheme was excluded, underscoring the advantage of jointly modeling second-scale physiology and minute-scale context.

### 7.7 Integrated summary

The analyses collectively support the following three primary conclusions. First, the hierarchical multimodal architecture enhances the discrimination and calibration of perioperative endpoints, providing narrow and informative uncertainty intervals, as demonstrated in Figure 3 through Figure 6 and in Table 5. Second, the sequence modeling recommendation module increases the off-policy value while maintaining minimal dose deviations and high clinician acceptance, yielding a greater net benefit across clinically relevant thresholds, as illustrated in Figure 7 through Figure 11 and in Table 6. Thirdly, performance and calibration are generalizable across institutions and subtypes, remaining robust in ablation studies, as shown in Figure 12 through Figure 17 and Figure 21. These attributes align with the requirements of anesthesia teams, which necessitate accurate, transparent, and reliable tools that are transferable across various sites and procedure types.

## 8 Discussion

Ortho PeriFT addresses critical clinical issues at the bedside, such as preventing hypotension, mitigating postoperative nausea and vomiting, and managing pain without oversedation. The model's capacity for early warning and suggesting constrained titrations of fluids, vasopressors, and anesthetic depth is clinically significant, given that both the depth and duration of blood pressure depression are linked to myocardial and kidney injury (2, 3). By calibrating risk estimates and incorporating uncertainty bands, clinicians can establish thresholds that align with service-level expectations and resource availability rather than relying on uncalibrated scores. For recovery, the system can personalize antiemetic prophylaxis by integrating documentation and intraoperative data streams with consensus risk factors, thereby complementing established guidelines and enabling decision curve analysis to quantify the net benefit in specific populations (5, 6, 28). This framework also supports goal-directed analgesic plans that balance efficacy and safety, consistent with multimodal analgesia recommendations (4).

The relevance of these findings to the day-to-day work of anesthetistsanaesthetists, surgeons, and perioperative teams is most clearly seen along the orthopedic orthopedic surgical pathway. Orthopedic orthopedic surgery is dominated by elective arthroplasty, fragility fractures, and major spine procedures in older, often comorbid patients, where small differences in haemodynamic stability, antiemetic strategy, and acute pain control translate into measurable differences in mobilizationmobilisation, length of stay, and complication rates. In the preoperative clinic, the calibrated multimodal risk estimates produced by Ortho PeriFT can support more structured risk stratification: patients flagged as higher risk for intraoperative hypotension or significant postoperative pain can be triaged toward toward preoperative optimisation (for example, anemia anemia correction, frailty assessment, or revision of antihypertensive regimens), discussion of regional versus general anesthesiaanaesthesia, and pre-emptive booking of higher-acuity postoperative care. Intraoperatively, the early-warning component, which provides longer median lead times at matched false-alarm rates, gives the anesthetist anaesthetist an actionable window to intervene before significant arterial pressure decreases, while the conformal uncertainty bands explicitly indicate when the system itself is uncertain and should be ignored or supplemented by direct clinician judgement. The constrained recommendation module is designed to operate as a guideline-aware suggestion layer, surfacing fluid, vasopressor, and depth adjustments that are within accepted dose and rate limits and can be accepted, modified, or rejected by the responsible anesthetistanaesthetist. Postoperatively, the per-patient PONV and acute pain risk estimates can be integrated into PACU handover and ward planning so that antiemetic choice, opioid-sparing strategies, and the intensity of postoperative observation are tailored to the individual patient rather than applied uniformly. Across these stages, Ortho PeriFT is therefore best understood not as an autonomous system but as a perioperative decision-support layer that augments existing assessment, planning, intraoperative management, and postoperative care pathways for orthopedic orthopedic patients.

Transformers are particularly well-suited for perioperative decision support for several reasons. First, long context attention links second-scale waveform patterns with minute-scale numerics, medication events, and narrative text without truncating clinically important history (15–17). Second, cross-modal fusion over an efficient latent array allows physiological streams, documentation, and optional imaging prompts to inform each other while preserving temporal order, thereby enhancing discrimination and calibration compared to single-stream baselines. Third, self-supervised pretraining leverages the abundance of unlabeled perioperative signals available in VitalDB, INSPIRE, MOVER, and large hospital or ICU corpora, resulting in robust encoders that transfer across sites and orthopedic subtypes (18–21). Combined with conformal prediction for calibrated intervals, the architecture supports decisions that are both data-rich and risk-aware (26).

This study has several limitations. First, documentation practices differ across institutions and over time, potentially introducing hidden confounding factors and label noise, particularly concerning pain scores and recovery symptoms. Second, site-specific practice patterns and device ecosystems may influence action availability and sampling frequency, posing challenges to deployment without meticulous calibration and governance. Third, the connection between preoperative imaging and intraoperative physiology is indirect and may reflect confounding by indication; thus, imaging prompts should be considered as optional contexts rather than determinants. Fourth, offline reinforcement learning depends on logged behavior; even with behavior regularization and action masks, off-policy estimators can be biased if support is limited or if the covariate shift is substantial (29, 30). Fifth, although the early-concatenation multimodal transformer and the late-fusion ensemble were introduced as conceptually appropriate multimodal comparators, the present manuscript does not report standalone numerical performance for these two new baselines; consequently, the claim that Ortho PeriFT outperforms the strongest comparator cannot be independently evaluated against these specific multimodal baselines from the tables in their current form, and per-baseline numerical results for the early-concatenation transformer and the late-fusion ensemble will be provided in a follow-up extension of this work. Finally, although conformal methods offer distribution-free guarantees under exchangeability, shifts in monitoring density and medication formularies can compromise coverage unless the calibration is updated (26).

The immediate path forward is prospective but intentionally conservative. A silent deployment phase should validate the calibration, lead time, and alarm workload without affecting care. This phase is succeeded by clinician-in-the-loop usage, where recommendations are presented as suggestions and acceptance or override rationales are recorded to refine policies. Public reproducibility is equally important. We intend to release a code that automatically curates orthopedic cohorts from VitalDB, INSPIRE, and MOVER with fixed splits, harmonized variable maps, and precise label definitions, enabling external groups to audit the results and conduct head-to-head benchmarks. Table 7 delineates a pragmatic evaluation plan, including safety guardrails, operating points derived from decision curves, and governance necessary for integration into clinical workflows (18–20, 28).

**Table 7:** Prospective evaluation blueprint for Ortho PeriFT.

Aspect	Silent deployment	Clinician-in-the-loop	Data and governance
Primary endpoints	Hypotension burden, PONV, high PACU pain	Same endpoints with acceptance and override logging	Cohorts curated from VitalDB, INSPIRE, MOVER with fixed splits (18–20)
Calibration and uncertainty	ECE, NLL, conformal coverage and width by site and subtype	Real-time calibration drift monitor with scheduled recalibration	Pre-registered thresholds and recalibration schedule approved by QA
Utility and workload	Decision curves, alert lead time at fixed false alarm rates	Acceptance rate, dose deviation, time-to-action, alarm fatigue audits	Human factors review and weekly safety huddles
Policy safety	Offline OPE WIS and DR, guardrail violations zero tolerance	Action masks enforced; abstain when intervals exceed width limit	Change control with rollback and audit trail
Success criteria	Non-inferior calibration vs baseline, higher net benefit, manageable alerts	Maintained safety, stable acceptance, no increase in rescue therapy rates	Independent data safety board review

The silent phase precedes the clinician-in-the-loop. Operating points are chosen by maximizing net benefit on internal development data and locked before external evaluation (28).

The results collectively endorse a cautiously optimistic view. Ortho PeriFT enhanced both discrimination and calibration for orthopedic anesthesia endpoints, provided uncertainty estimates that facilitated abstention, and proposed dose-constrained actions that increased counterfactual utility across multiple estimators. The remaining tasks are translational: demonstrating stability in silent trials, refining human-AI interfaces that align with clinician workflows, and establishing a public benchmark to ensure that advancements are measurable and reproducible across different centers.

## 9 Ethics, governance, and reporting

All datasets utilized in this study are publicly accessible under explicit data use agreements or access terms and were managed according to their governance policies. VitalDB and MIMIC-IV are distributed via PhysioNet under credentialed access, necessitating a data use agreement, training, and attestation. Both resources are de-identified in compliance with applicable standards and are accompanied by comprehensive documentation detailing the removal of protected health information (18, 21). INSPIRE is released on PhysioNet with project-specific permissions and provenance records, including cohort construction and variable dictionaries suitable for reproducible research (19). MOVER is available through a public access process administered by its stewards and includes operating room vitals and events linked to electronic health records after deidentification (20). For all datasets, we processed only de-identified records, maintained version-controlled pipelines, and exported shareable manifests and configuration files to enable third parties to reproduce preprocessing, splits, and label derivations without access to any identifiers.

Governance structures emphasize traceability and the principle of least privilege. Access to raw data was conducted using dedicated accounts linked to completed training and signed agreements. All experiments were performed in containerized environments with immutable logs of code, parameters, and random seeds. We preserved an auditable lineage from raw inputs to analysis-ready tensors, including unit normalization and clock alignment. Model artifacts are stored with checksums and metadata that capture dataset versions and exclusion criteria, supporting external audits and facilitating rollbacks if necessary.

Bias and harm assessments were conducted at three levels. First, we evaluated calibration and discrimination across age, sex, and body mass index strata and reported subgroup differences with confidence intervals, adhering to recommendations for fairness evaluation in clinical prediction (71). Second, we performed counterfactual reweighting analyses to mitigate confounding factors when comparing subgroups. Third, we instituted prospective guardrails for deployment readiness that require zero tolerance for guideline violations in the action space and mandate abstention when the conformal intervals exceed the predefined widths. These procedures aim to mitigate both allocative harm and workflow burden while ensuring clinician involvement.

The model documentation follows the emerging best practices. We release model cards that specify the intended use, out-of-scope settings, input requirements, training data composition, performance and calibration across subgroups, uncertainty behavior, and known failure modes (72). Reporting adheres to established guidelines for clinical artificial intelligence studies; in particular, we map our evaluation to items emphasized by CONSORT AI and SPIRIT AI, including dataset provenance, handling of missingness, external validation, and risk of bias considerations relevant to future prospective studies (73). Together, data governance, bias auditing, model documentation, and transparent reporting provide a foundation for safe iteration toward clinician-in-the-loop use while preserving reproducibility and accountability.

## 10 Conclusion

Orthopedic anesthesia necessitates precise and timely decision-making that balances hemodynamic stability, patient comfort, and safety. This study introduces Ortho PeriFT, a hierarchical multimodal transformer designed to deliver calibrated predictions, constrained therapy recommendations, and continuous monitoring within a unified framework. By aligning representation learning with clinical timescales and integrating waveforms, numerical data, medications, events, and text, the model enhances discrimination and precision recall for hypotension, postoperative nausea and vomiting, and significant postanesthesia care unit pain. It also reduces the calibration error and negative log likelihood, provides narrow uncertainty intervals that support abstention, and suggests dose-constrained actions that enhance counterfactual utility without breaching established guardrails.

Beyond aggregate accuracy, Ortho PeriFT advances practical readiness in clinical settings. Early warning analyses demonstrate extended lead times at matched false-alarm rates, potentially creating actionable opportunities for intervention. Subgroup and subtype evaluations revealed stable calibration across demographic strata and orthopedic procedures, supporting equitable performance in diverse operating room contexts. Attribution maps and prototype retrieval offer case-based rationales that align with clinical reasoning, potentially enhancing trust and facilitating oversight.

These findings suggest that a single safety-aware transformer can unify perioperative prediction and decision support for orthopedic surgery. The subsequent step involves careful prospective evaluation with silent deployment, followed by clinician-in-the-loop use, which logs acceptance and overrides to refine policies. If validated, this approach could assist anesthesia teams in delivering more consistent hemodynamic control, personalizing antiemetic and analgesic strategies, and streamlining monitoring, ultimately improving recovery quality and resource utilization in orthopedic care. In practical terms, we anticipate the most immediate value for routine orthopedic orthopedic pathways to lie in three areas: structured preoperative risk stratification of older, comorbid patients presenting for arthroplasty or fragility-fracture surgery; intraoperative early-warning and guideline-compliant titration support for the responsible anesthetistanaesthetist; and individualized individualized postoperative antiemetic and multimodal analgesic planning that complements enhanced recovery protocols. The ultimate intent is that anesthetistsanaesthetists, surgeons, and perioperative teams retain full clinical authority while gaining a calibrated, auditable risk signal tailored to the specific demands of orthopedic orthopedic surgery.

## Data Availability

The original contributions presented in the study are included in the article/supplementary material, further inquiries can be directed to the corresponding author.
